# Entry of *Yersinia pestis* into the Viable but Nonculturable State in a Low-Temperature Tap Water Microcosm

**DOI:** 10.1371/journal.pone.0017585

**Published:** 2011-03-16

**Authors:** David R. Pawlowski, Daniel J. Metzger, Amy Raslawsky, Amy Howlett, Gretchen Siebert, Richard J. Karalus, Stephanie Garrett, Chris A. Whitehouse

**Affiliations:** 1 CUBRC, Inc., Buffalo, New York, United States of America; 2 United States Army Medical Research Institute of Infectious Diseases, Frederick, Maryland, United States of America; 3 Department of Microbiology and Immunology, State University of New York at Buffalo, Buffalo, New York, United States of America; University of Osnabrueck, Germany

## Abstract

*Yersinia pestis*, the causative agent of plague, has caused several pandemics throughout history and remains endemic in the rodent populations of the western United States. More recently, *Y. pestis* is one of several bacterial pathogens considered to be a potential agent of bioterrorism. Thus, elucidating potential mechanisms of survival and persistence in the environment would be important in the event of an intentional release of the organism. One such mechanism is entry into the viable but non-culturable (VBNC) state, as has been demonstrated for several other bacterial pathogens. In this study, we showed that *Y. pestis* became nonculturable by normal laboratory methods after 21 days in a low-temperature tap water microcosm. We further show evidence that, after the loss of culturability, the cells remained viable by using a variety of criteria, including cellular membrane integrity, uptake and incorporation of radiolabeled amino acids, and protection of genomic DNA from DNase I digestion. Additionally, we identified morphological and ultrastructural characteristics of *Y. pestis* VBNC cells, such as cell rounding and large periplasmic spaces, by electron microscopy, which are consistent with entry into the VBNC state in other bacteria. Finally, we demonstrated resuscitation of a small number of the non-culturable cells. This study provides compelling evidence that *Y. pestis* persists in a low-temperature tap water microcosm in a viable state yet is unable to be cultured under normal laboratory conditions, which may prove useful in risk assessment and remediation efforts, particularly in the event of an intentional release of this organism.

## Introduction

*Yersinia pestis* is a gram-negative bacterium that is the etiological agent of plague, a rapidly progressing, serious illness in humans. The plague bacterium has given rise to at least three major pandemics throughout history and still causes several thousands of human cases per year worldwide [1]. This includes the infamous Black Death in Europe where approximately a quarter of the population was killed within a span of two years. Given the highly pathogenic nature of *Y. pestis*, the organism is regarded as a potential bioterrorism agent. *Yersinia pestis* is a zoonotic pathogen that is normally transmitted by the bite of an infected flea and is generally thought to exist in enzootic cycles that involves transmission between rodents and their fleas. The disease can, however, spread to other, more susceptible animals causing rapidly spreading die-offs or epizootics. Most human cases are believed to occur during these epizootic periods when highly susceptible hosts die in large numbers and their fleas are forced to parasitize other hosts, including humans [2].

In the environment, *Y. pestis* is thought to survive for only short periods of time outside a host While this is likely true in most cases a growing body of evidence suggests that *Y. pestis* can survive without a host for extended periods under certain environmental conditions while, in many cases, retaining infectivity [3], [4], [5], [6]. For instance, more than a century ago, Alexander Yersin described isolating *Y. pestis* from the soil of a house where the inhabitants had died of plague [7]. Nearly 70 years later, in controlled experiments, Mollaret showed that *Y. pestis* remained infective in soil for nearly one year [3]. Other, more recent work by Ayyadurai *et al*. demonstrated that *Y. pestis* remained viable and fully virulent after 40 weeks in soil [8]. In addition to survival in soil, Rose *et al*. showed that *Y. pestis* could survive for limited periods of time (i.e., hours) on a variety of environmental surfaces [9]. More relevant to this study, in 1897, Wilm determined the survival time for *Y. pestis* in tap water to be 16 days [10]. A more recent study by Torosian has shown that many *Y. pestis* strains persist in bottled drinking water for over 100 days [11]. Additionally, Torosian has shown that, given the proper nutrient requirements, *Y. pestis* can not only persist but actually multiply at 4°C. These works indicate that *Y. pestis* can persist in atypical environments raising the questions of how the organism is able to survive without its typical hosts and under what conditions is *Y. pestis* likely to persist.

There is a growing list of pathogenic gram-negative bacteria that survive adverse conditions by entering the VBNC state [12]. Some of the known conditions that induce entry into the VBNC state are cold, starvation, chlorination, and osmotic stress [12], [13]. Reports indicate that many enteric bacteria can be found in the VBNC state in treated wastewater or induced into this state in drinking water [14], [15], [16]. The two closest pathogenic relatives of *Y. pestis*, namely *Y*. *enterocolitica* and *Y. pseudotuberculosis*, have been shown to enter the VBNC state [17], [18], [19], [20]. In addition, many other bacterial pathogens, including *Francisella tularensis*, *Vibrio cholerae*, and *Escherichia coli* have been shown to enter the VBNC state [12], [21].

The objective of this study was to determine if *Y. pestis* could enter into the VBNC state after prolonged exposure in water at 4°C. We examined these cells for viability by several methods, including determination of cell membrane integrity, incorporation of radiolabeled amino acid, and DNaseI protection of genomic DNA. Finally, scanning and transmission electron microscopy (SEM and TEM) was used to visually compare nonculturable *Y. pestis* with those that were actively growing. This work provides new information on the biology of *Y*. *pestis* and could have a major impact on risk assessment and remediation efforts, particularly in the event of an intentional release of this pathogen.

## Results

### Persistence of *Y. pestis* in water

**Figure 1:**
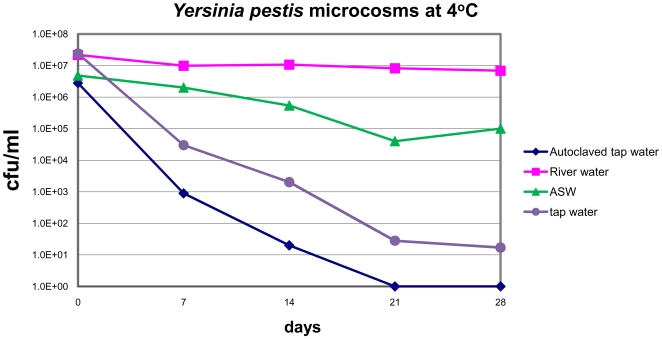
Culturability of *Y. pestis* on TSA II plates as a function of culture time in different water based microcosms. The *Y. pestis* in the autoclaved tap water microcosm (♦) became unculturable after 21 days.

Figure 1 depicts the number of colonies of *Y. pestis* per milliliter as a function of incubation time in water at 4°C. Exposure to chlorinated water typically results in a rapid decrease in culturability as a result of cell death. We therefore tested the free and total chlorine levels of our tap water both before and after autoclaving to assess chlorine's potential role in this experimental system. After autoclaving the levels of both free and total were below the limits of detection (>0.1 mg/L). The initial inoculum of 3×10^6^ cfu/mL declined to below 0.1 cfu/mL within 21 days. The non-autoclaved tap water that had detectable chlorination (0.3 mg/L free chlorine and 0.6 mg/L total chlorine) led to a drastic (10^6^) yet incomplete loss of culturability over a 28 day period. The incomplete loss of culturability when compared to the autoclaved microcosm is likely due to the larger initial inoculum size (approximately 2×10^7^ cfu/mL, almost 10-fold higher). In both tap water microcosms however, these results indicated that there was a loss of 10^6^ cfu/mL following 21 days of incubation. These data are almost identical to those determined by Wilm over 100 years ago who reported a loss of culturability in tap water after 16 days [10]. In direct contrast, however, *Y. pestis* incubated in artificial sea water or sterilized river water at 4°C exhibited a lesser extent of decline in culturability after the 28 day period (Figure 1).

### *Y. pestis* Metabolic Activity Determination

#### Membrane maintenance

**Figure 2:**
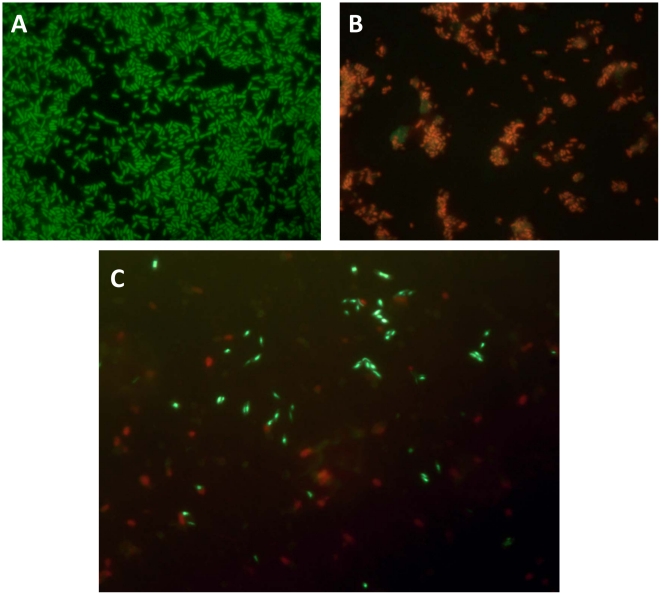
Determination of *Y. pestis* viability using the LIVE/DEAD® *Bac*Light™ Bacterial viability kit. Panel A is a representative field of a single colony grown for 48 hours on TSA II agar at 37°C and resuspended in PBS. Panel B is a representative field of a single colony grown for 48 hours on TSA II agar at 37OC and resuspended in PBS and heat-killed for 10 minutes at 95°C. Panel C is a representative field of the microcosm after 46 days at 4°C. Cells fluorescing green are considered alive while red fluorescing cells are considered dead.

Exposure to chlorinated water typically results in a rapid decrease in culturability as a result of cell death however, chlorinated water is also implicated in entry into the viable but non-culturable (VBNC) state [13]. The dramatic loss in culturability of both tap water cultures led us to test for the presence of the viable bacteria despite its inability to grow on laboratory media. The viability of bacteria that are not culturable can be determined by measuring membrane integrity or metabolic activity. We tested the autoclaved tap water microcosm for signatures of membrane integrity by using the LIVE/DEAD® *Bac*Light™ bacterial viability kit. This kit is a dual nucleic acid dye system that uses the green-fluorescent dye, SYTO®-9, and the red-fluorescent stain, propidium iodide. SYTO®-9 traverses intact membranes; whereas, propidium iodide cannot. Therefore, cells with intact membranes (considered to be alive) fluoresce green, while those with damaged membranes (considered to be dead) fluoresce red. Figure 2, Panel C shows a representative field of non-culturable *Y. pestis* cells after 46 days in low-temperature autoclaved tap water stained with the LIVE/DEAD® *Bac*Light™ and viewed using epifluorescence. The figure shows approximately half of the cells fluorescing green, indicating these cells have an intact cellular membrane which is indicative of live cells. The red fluorescing cells lacked cell membrane integrity and represent dead cells. These data suggest that a significant number of the initial inoculum of 3×10^6^ maintain membrane integrity, which is indicative that the cells are viable. In light of the data recently published by Torosian *et al*. showing that *Y. pestis* actively reproduces at 4°C, our data also suggests that *Y. pestis* is able to survive for long periods of time in water [22].

#### DNase I protection assay

The evidence indicating the maintenance of membrane integrity led us to perform a DNase I protection assay as a confirmation. The DNase I protection assay is a measure of cellular integrity as intact membranes protect genomic DNA from digestion by exogenous nucleases whereas ruptured or leaky membranes cannot. Table 1 contains the results of a DNase I protection assay performed on actively growing and the non-culturable *Y. pestis* from the low-temperature, autoclaved tap water microcosm. Quantitative real-time PCR was used to obtain the cycle number at the threshold level of log-based fluorescence (Ct value) which is proportional to the amount of total intact target DNA present in the sample. Samples were treated with and without DNase I digestion prior to the qRT-PCR step. In addition, controls where cells were heat killed to disrupt the bacterial cellular membranes thus allowing the enzyme access to the cell's genomic DNA were performed.

**Table 1:** DNase I protection assay of non-culturable *Y. pestis* cells from the autoclaved tap water microcosm compared to actively growing *Y. pestis.*

SAMPLE	Average Ct (SD)
**Non-culturable ** ***Y. pestis***	
Heat-killed (no DNase I)	20.32 (0.03)
Heat-killed (24-h DNase I)	26.14 (0.29)
Not heat-killed (no DNase I)	19.98 (0.3)
Not heat-killed (24-h DNase I)	21.7 (0.26)
**Actively growing ** ***Y. pestis***	
Heat-killed (no DNase I)	26.97 (0.17)
Heat-killed (24-h DNase I)	30.62 (0.4)
Not heat-killed (no DNase I)	24.56 (0.4)
Not heat-killed (24-h DNase I)	26.07 (0.14)

Results indicate that samples containing non-culturable cells that were heat-treated and then exposed to DNase I for 24 hours had a significant (∼6 cycle) decrease in Ct values compared to those without DNase I treatment (Table 1). These data indicate that heat-killed cells lost membrane integrity, allowing the nuclease to degrade the cell's genomic DNA. On the other hand, samples containing non-culturable cells that were not heat-treated, but were likewise exposed to DNase I for 24 h, had only a modest (less than 2 cycles) decrease in their Ct values. These data suggest that non-culturable bacteria that were not exposed to heat treatment displayed a greater propensity to protect genomic DNA from DNase I digestion, indicating these cells maintain membrane integrity. Similar differences in Ct values were observed for a control group of actively growing *Y. pestis* (Table 1) suggesting that the non-culturable bacteria are indeed alive.

#### Radiolabeled amino acid incorporation

**Figure 3:**
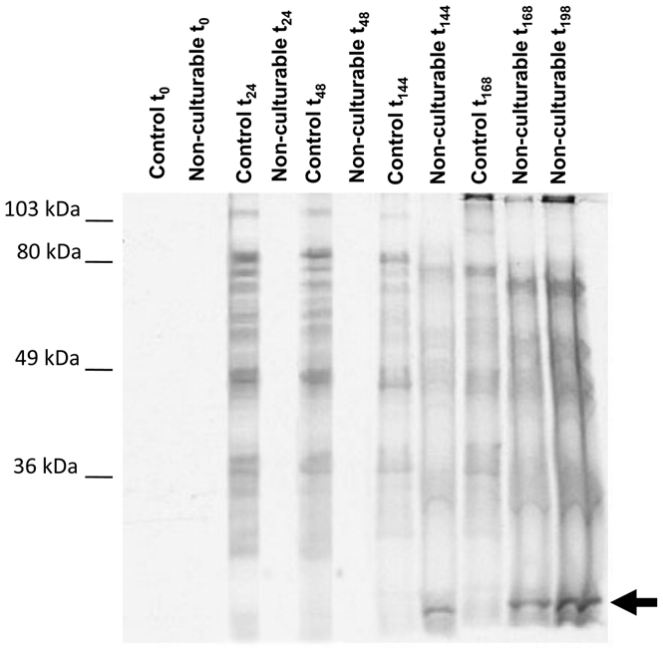
Autoradiogram of an SDS-PAGE gel following radiolabeled amino acid uptake by the non-culturable *Y. pestis* in an autoclaved tap water microcosm. Actively growing control cells demonstrate uptake and incorporation within 24 hours whereas the cells within the nonculturable microcosm did not incorporate radiolabel until later (144 hours). The nonculturable cells incorporated amino acids into different size proteins than the actively growing control group as indicated by the arrow.

Uptake of radiolabeled amino acids is another means to assess metabolic activity in non-culturable bacteria [23], . We used a method similar to Rahman, Roszak and Colwell to determine if non-culturable *Y. pestis* in autoclaved tap water were able to incorporate amino acids into newly translated proteins, which would indicate viability based on active metabolism. A mixture of S^35^–labeled methionine and cysteine was incubated with the nonculturable and actively growing control cultures for up 198 hours. At specified time points, samples were removed and protein directly separated by SDS-PAGE. The data presented in Figure 3 demonstrate that the non-culturable cells incorporate radiolabeled amino acids as evidenced by the banding pattern on this autoradiograph. As one would expect, amino acid incorporation was appears to be slower for the non-culturable cells compared to the normal cells. For example, quantification of each lane indicated that it took approximately 144 hours for the nonculturable cells to reach comparable incorporation levels to control after 24 hours. This result can be interpreted in multiple ways. One possibility is that metabolism in the non-culturable microcosm has considerably slowed leading to less uptake. Another possible interpretation is that there are fewer live cells in the microcosm leading to less incorporation over a similar time period. The final possibility, and most likely, is a combination of the original two interpretations. Due to our inability to accurately calculate the number of cells in the VBNC state, we are unable to distinguish between these possibilities. A second interesting observation from these data is that there was a different labeled protein profile between the non-culturable and actively growing cells, including a low-molecular weight protein that appears to be unique to the non-culturable cells (arrow, Fig. 3). Specific identification of the differentially expressed bands was beyond the scope of this study, but would be an interesting avenue for further research.

### Morphological changes of the VBNC *Y. pestis*

**Figure 4:**
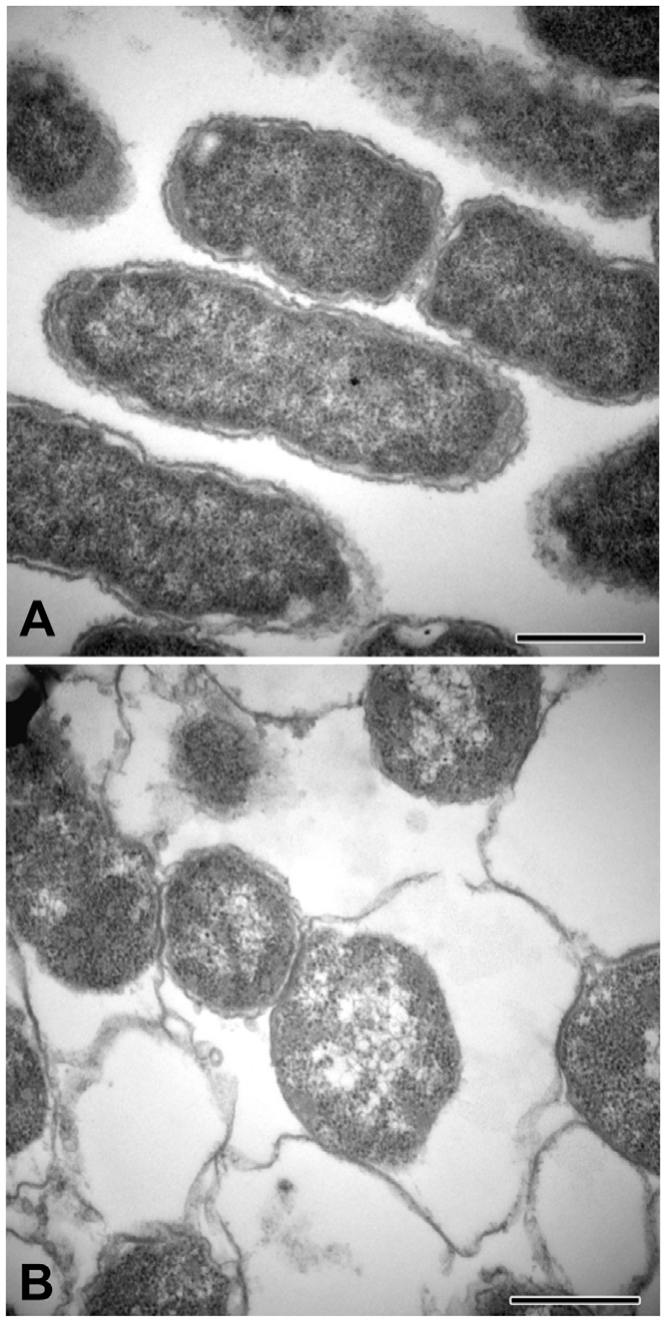
Transverse section of a transmission electron micrograph of actively growing (Panel A) and nonculturable (Panel B) *Y. pestis*. The arrow in panel B points to the enlarged periplasmic space. Magnification 10,000X, bar equals 0.5 microns.

**Figure 5:**
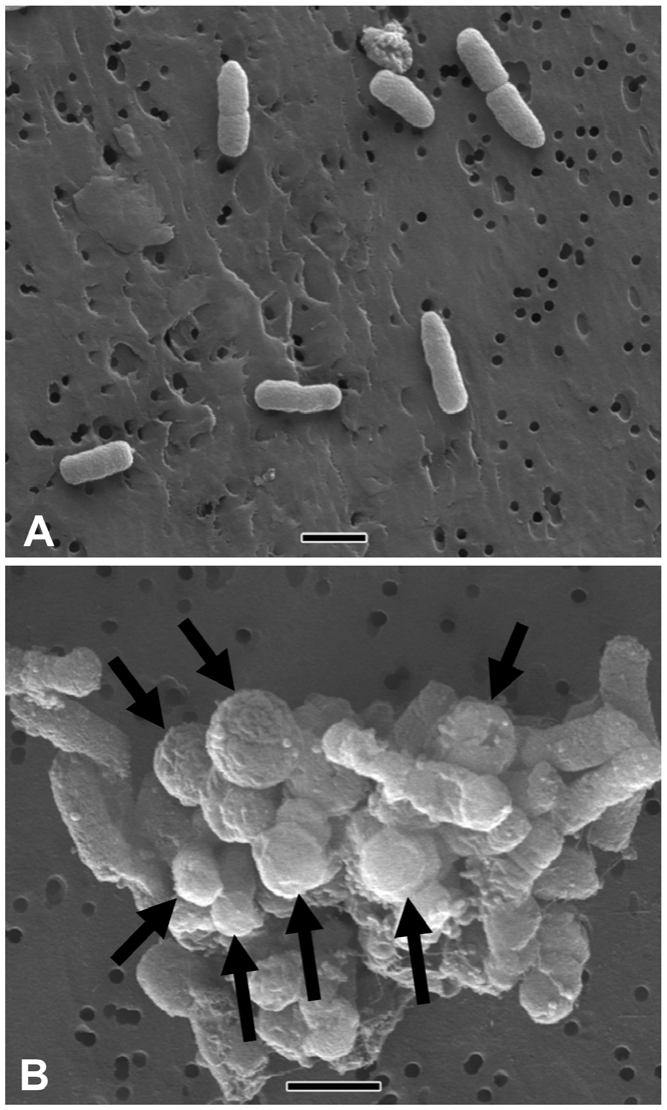
Scanning electron micrograph of actively growing (Panel A) and nonculturable (Panel B) *Y. pestis*. The coccoid shape of the cells in the tap water microcosm is apparent (Panel B arrows). Magnification in panel A is 5,000X and 10,000X in panel B, bar equal to 1.0 micron.

In addition to the three sets of data that suggest metabolic activity discussed above, the non-culturable *Y. pestis* exhibited several morphological and ultrastructural changes as observed by both transmission electron microscopy (TEM) and scanning electron microscopy (SEM). Figure 4 shows representative microscopic fields of laboratory grown (Panel A) and non-culturable *Y. pestis* (Panel B) cells under TEM. Most notably, laboratory grown cells had tight membrane junctions (Fig. 4A); whereas, the periplasmic space was greatly increased in the non-culturable cells (Fig. 4B). Additionally, the cytoplasm of the non-culturable cells has condensed into a small rounded cytosol with a volume of approximately half that of the laboratory grown cells. Figures 5A and 5B depict SEM micrographs of actively growing and non-culturable cells, respectively. The most apparent morphological change in the non-culturable cells was a shift from rod-shaped to a more cocci-like cell shape. Although there are definite morphological differences between non-culturable and cultured *Y. pestis* as observed by electron microscopy, it is difficult to make any conclusions since we have no way to determine live from dead cells using this technique.

### Resuscitation from the non-culturable state

The existence of a VBNC state in bacteria argues that there must be a mechanism to exit the VBNC state to resuscitate growth otherwise it is a dead-end state. We therefore attempted to resuscitate our non-culturable *Y. pestis*. Resuscitation was attempted with three types of liquid media using a method meant to determine the most probable number (MPN) estimate of viable cells [26]. In addition, we attempted to resuscitate cells in presence and absence of sodium pyruvate following the example of Lleo *et al.* [27]. The three media chosen for an initial resuscitation attempt were chlorine depleted tap water shifted to room temperature and 37°C, 1/8 Brain Heart Infusion (BHI) and 2% Fetal Bovine Serum (FBS) (ATCC, Manassas, VA, USA). We chose these media as it has been shown that temperature shifting and decreased nutrient load are effective tools to resuscitate certain VBNC bacteria [12], [28]. Table 2 shows that only the cultures where the microcosm was diluted in 1/8 BHI media (Difco™, BD Diagnostics, NJ, USA), showed turbidity after 72 hours at room temperature. The other media did not support bacterial growth. These data indicate a lack of the proper resuscitation factor in water and FBS. Despite the lack of turbidity in water and FBS, plating was performed to acquire an accurate estimate in the event of low-level resuscitation. No colonies were observed to grow on solid media from any of the liquid cultures that were not turbid. These results indicated that there is a particular factor(s) in 1/8 BHI that *Y. pestis* may require or utilize for exit from a non-culturable state however the amount of outgrowth was small compared to the number of predicted viable cells. This result suggests that there may be preferred factor(s) for *Y. pestis* outgrowth that are not in BHI or that only a small fraction of the non-culturable population is able to re-enter a growth phase.

**Table 2:** Most probable number, ten tube test used to calculate the number of viable cells in the autoclaved non-culturable microcosm.

	tubes displaying growth by turbidity				
Sample	No dilution	10^−1^	10^−2^	10^−3^	CFU/mL
Control (Tap Water)	0	0	0	0	0
1/8 BHI	10	10	4	1	5.9
2% FBS	0	0	0	0	0
Tap water + 2 mg/mL sodium pyruvate	0	0	0	0	0
1/8 BHI + 2 mg/mL sodium pyruvate	10	9	3	1	2.9
2% FBS + 2 mg/mL sodium pyruvate	0	0	0	0	0

## Discussion

*Yersinia pestis* has been the cause of some the most devastating disease epidemics in human history, including at least three world-wide pandemics [1]. The recent classification of this bacterium as a potential agent of bioterrorism has spurred increased interest in its ecology and persistence. At least two studies have been reported showing that *Y. pestis* can survive for extended periods (as much as 40 weeks) in soil and maintain its virulence [3], [4] [8].

The objective of this study was to examine the mechanism of persistence of *Y. pestis* in tap water, an environment not normally studied with regards to this bacterium, yet one implicated in driving bacteria to into the VBNC state [16]. We detected 0.3 mg/L of free chlorine in fresh tap water and were unable to detect chlorine in autoclaved tap water. There is very little data regarding *Y. pestis* survival is such environments; however, Rose *et al.* showed that monochloramine levels typically found in municipal tap water decrease the number of culturable *Y. pestis* by 2-log10 in under 2 hours [29]. In addition to tap water, we monitored the persistence of *Y. pestis* in two other common waters, river water and sea water for comparison. In many cases, bacteria that are incubated at low temperatures in nutrient restricted environments enter a state where they become non-culturable by normal laboratory methods known as the viable but non-culturable (VBNC) state [30]. In our initial experiments, we simply examined the persistence of viable (as measured by colony outgrowth) *Y. pestis* in the aforementioned water environments, namely, artificial seawater, sterilized natural river water, and tap water at 4°C. We chose 4°C as our initial temperature because it has been implicated in driving a number of bacteria, including close relatives of Y. pestis into the VBNC state [19]. As seen in Figure 1, *Y. pestis* in tap water had a 6-log reduction in culturable cells, and the autoclaved tap water microcosm became non-culturable (after 21 days). There are at least three possible reasons for this rapid decline in culturability. One obvious reason is that tap water has far fewer nutrients compared to the other environments. Another potential reason may be the rapid alteration in osmotic stresses placed on *Y. pestis* during transfer from a nutrient rich media to tap water. Lastly, the presence of chlorine (0.3 mg/L and below 0.1 mg/L) in the tap water could induce *Y. pestis* into a non-culturable state. Past studies suggest that the later possibility is the most likely, for example, Oliver *et al*. showed that *E. coli* and *Salmonella typhimurium* could be induced into a non-culturable state after chlorination of wastewater [16]. It is interesting to note that between the autoclaved and non-autoclaved tap water microcosms, the one with less available chlorine reached a state of non-culturability first. We surmise this result was due to the larger inoculum size than the autoclaved microcosm.

The timing that our *Y. pestis* microcosm took to become non-culturable appears to be similar to other gram-negative pathogens. For example, Mizunoe *et al*. demonstrated that *E. coli* O157:H- strain E 32511/HSC became non-culturable in sterilized distilled water microcosms at 4°C after 21 days [31]. Likewise, Linder and Oliver reported that *Vibrio vulnificus* entered the VBNC state after 24 days of incubation [32]. In a recent study, Du *et al*. showed that the fish pathogen, *Edwardsiella tarda*, became non-culturable after 28 days incubation at low temperature [33]. Thus, our data showing that *Y. pestis* became non-culturable after 21 days incubation in sterilized tap water are consistent with the previously published data for other gram-negative bacterial pathogens.

One possible mechanism for the persistence and survival of *Y. pestis* in the environment may be entry into the VBNC state. The VBNC state is defined as a condition where bacteria fail to grow under normal laboratory-defined growth conditions, yet the cells are still alive and maintain metabolic activity [12]. There is a growing list of pathogenic gram-negative bacteria that survive adverse conditions by entering the VBNC state [12], [30]. Some of the known conditions that induce entry into the VBNC state are cold, starvation, chlorination, and osmotic stress [12], [13]. Reports indicate that many enteric bacteria can be found in the VBNC state in treated wastewater or induced into this state by chlorinated water [14], [15], [16]. Studies indicate that many bacterial pathogens, including *Francisella tularensis*, *Vibrio cholerae*, and *Escherichia coli* enter the VBNC state [12], [21]. In addition, the two closest pathogenic relatives of *Y. pestis*, namely *Y*. *enterocolitica* and *Y. pseudotuberculosis*, also enter the VBNC state [17], [18], [19], [20], [34]. Therefore it should not be unexpected to find evidence that *Y. pestis* be capable of entering this state as well.

Bogosian *et al*. has shown that the addition of sodium pyruvate partially alleviates the loss of *Vibrio vulnificus* culturability under similar conditions to those used in this study [35]. In addition, Lleo *et al*. observed a moderate (2-fold) increase in *Enterococcus faecalis* culturability with the addition of sodium pyruvate. These works have lead to the hypothesis that the addition of sodium pyruvate provides a more accurate culturable cell count by allowing reactive oxygen species (ROS) sensitive cells to grow on nutrient rich media [27]. We tested this hypothesis by plating our cells with and without sodium pyruvate. We did not see a difference in cell numbers nor did the time to loss of culturability vary with the addition of sodium pyruvate, that is, sodium pyruvate did not extend culturability beyond 21 days.

Our results provide evidence of cell viability when not-culturable using several independent criteria, including membrane integrity and active metabolism. We demonstrated retention of cell membrane integrity using the LIVE/DEAD^®^ *Bac*Light™ viability staining which functions based on the ability of live cells to prevent nucleic acid specific dye from entering the cell (Fig. 3). The majority of *Y. pestis* cells fluoresced green after 46 days (25 days after first becoming non-culturable) in the low temperature tap water microcosm indicating an intact cell membrane that is capable of preventing dye entry. We provided additional evidence of membrane integrity by demonstrating that the genomic DNA of non-culturable *Y. pestis* were protected from DNase I digestion, and this protection was abolished by heat-killing the cells prior to DNase treatment. These data indicate that the non-culturable *Y. pestis* from the tap water microcosm were protected from DNase I treatment, and thus possessed active cellular membrane maintenance and were indeed viable cells (i.e., VBNC). A third line of evidence demonstrating cell viability was the ability of non-culturable *Y. pestis* to take up exogenous radiolabeled amino acids and incorporate them into newly synthesized protein (Fig. 3). The ability to incorporate exogenous amino acids implies that the non-culturable *Y. pestis* retain an active metabolite sensing/uptake system as well as active protein translation. We also demonstrated a different protein profile between non-culturable and vegetative cells, including the production of a low-molecular weight protein that appears to be uniquely expressed in the non-culturable cells under the conditions that we used (arrow, Fig. 3). While these data are consistent with other reports showing differential protein expression in VBNC cells, more work should be performed, including whole proteome analysis using 2-D gel electrophoresis, to further characterize *Y. pestis* [36].

Morphological changes of the non-culturable *Y. pestis* cells were identified using TEM and SEM. Under TEM, the cells from the non-culturable microcosm exhibited distinctive enlargement of their periplasmic space, and nuclear condensation (Figure 4). These changes are consistent with published observations for VBNC *V. cholerae* O1 and O139 [37]. Furthermore, cell morphology changed from rod-shaped to coccoid and cells were in clumps or clusters as observed using SEM (Figure 5). This is in agreement with the results reported for other bacteria that enter the VBNC state [33], [37], [38], [39]. The significance of these changes is not completely understood, but may be a survival strategy to minimize cell maintenance requirements during periods of starvation.

While the data presented above offer strong evidence for entry and persistence in the VBNC state, resuscitation from the VBNC state is a requisite for the existence of such a state. We therefore explored resuscitation parameters for the non-culturable *Y. pestis* microcosms. We were able to demonstrate low levels of resuscitation as illustrated by outgrowth in 12.5% (1/8) BHI broth. The low level of resuscitation result is not unexpected as the resuscitation factor for many VBNC bacteria is typically a specific signal or molecule often missing from laboratory based media. For example, *Legionella pneumophila* is resuscitated only in the presence of specific amoebae [40]. Similarly, the *Rpf* gene product of *Micrococcus luteus* is required by *Mycobacterium tuberculosis* to exit dormancy [41].

In conclusion, we have provided several lines of evidence that *Y. pestis*, the causative agent of plague, can enter into a non-culturable state upon exposure to low-temperature tap water, which is consistent with descriptions of the VBNC state [37], [38], [42], [43], [44]. Based on these results we are reporting the first evidence that *Y. pestis* enters into the VBNC state. While this finding provides significant information in the biology of this important human pathogen, it also has practical applications in risk assessment and remediation efforts related to persistence of plague bacilli in the environment, particularly in the event of an intentional release of the organism.

## Materials and Methods

### Bacterial strains, preparation of microcosms, and culturability assay

*Yersinia pestis* (Harbin 35 strain), obtained from the Unified Culture Collection at United States Army Medical Research Institute of Infectious Diseases, Fort Detrick, was used for the microcosm experiments. Single colonies of bacteria were picked from trypticase soy agar (TSA; Remel, Lenexa, KS) plates, transferred to 3 ml of heart infusion broth (HIB) (Difco™, BD Diagnostics, NJ, USA) supplemented with 0.2% xylose, and incubated overnight at 26°C. Overnight seed cultures were diluted 1∶1000 into 200 ml of fresh HIB with 0.2% xylose and incubated at 26°C until they reached mid-logarithmic phase of growth (∼20 h). The bacteria were harvested by centrifugation at 5000×g for 20 min at 4°C, washed twice, and resuspended in 10 ml of autoclaved and filter-sterilized (0.22 µm) tap water. To create each microcosm, 500 µl of washed cells were inoculated into 50 ml tap water in sterile 50 ml conical tubes (Corning) at a final concentration of approximately 10^7^ cells ml^−1^. Microcosms were maintained at 4°C without shaking until cells were no longer culturable. River water was obtained from the upper Niagara River, filtered and autoclaved. Artificial sea water was made using distilled, deionized water and Instant Ocean™ as per the manufacturer's instructions.

Culturability of *Y. pestis* in each microcosm was determined every 7 days by culture on non-selective TSA plates. Samples from the microcosms were serially diluted in sterilized tap water, spread in duplicate on plates, and incubated at 26°C for 48 h. When the culturable cell populations were less than 10 colony forming units (CFU) ml^−1^, a 10 ml aliquot of the microcosm was centrifuged at 5000×g to pellet the cells, which were resuspended in 1 ml of sterile tap water, and the entire 1 ml suspension plated on TSA. The bacteria were considered to be non-culturable when <0.1 CFU ml^−1^ of the culturable cells could be detected by plate count. Radical oxygen species sensitivity was monitored by adding sodium pyruvate to TSA plates, 250 µl of 1 mole l^−1^ prior to bacterial plating.

Tap water chlorination levels, pre and post sterilization, were determined using the Hach chlorine, free and total test kit as per manufacturer's instructions (cat. no. 2231−01, Hach, Loveland, CO, USA). In addition to our own chlorine measurements, water quality reports have been included as supplemental material (File S1 and File S2).

### LIVE/DEAD® *Bac*Light™ viability assay

Bacteria viability was determined using the LIVE/DEAD® *Bac*Light™ viability kit (Molecular Probes, Eugene, OR). One milliliter aliquots of 10-fold dilutions of the bacterial microcosms were stained with a 6 µl mixture (1∶1) of SYTO®-9 and propidium iodide and incubated for 25 min in the dark at room temperature. The stained bacteria were filtered onto pre-wet 25-mm, 0.2-µm pore size, black polycarbonate membrane filter using vacuum suction (Millipore) and washed twice with 2 ml of saline (0.85%). Filters were mounted onto glass slides with low-fluorescence immersion oil (type A, Cargille, Ceder Grove, NJ) and examined with an epifluorescence microscope (Nikon). Digital photographs were obtained using an Infinity 3 digital camera and its accompanying Infinity Capture software package.

### DNase I protection assay

Bacteria were harvested by centrifugation and resuspended in 1X DNase I buffer (New England Biolabs, MA, USA). Samples were divided into two aliquots, one being heated at 95°C for 15 min. to lyse the cells while the other was kept on ice to prevent cell lysis. Heat-treated samples were cooled to room temperature before the addition of enzyme. Four units of DNase I were added to each sample and incubated at 37°C for the times indicated in table 1. The enzyme was inactivated by heating to 85°C for 5 min in accordance with the manufacturer's recommendations. Both groups (heat killed and un-treated) of samples were then analyzed for the presence of genomic DNA by quantitative real-time PCR as described below.

### Quantitative real-time PCR

A previously validated quantitative real-time PCR assay that targets *Ypo*1670, a conserved chromosomal gene from *Y. pestis*, was used in these studies. The primer sequences were as follows: forward primer, 5′-GCCGACGAGATTATCCAAATTG-3′; reverse primer, 5′-AATGTGCCCCGACCCATA-3′; probe, 5′-AAAAGCGGTAGACTCC-3′. The PCR master mix contained 20 mM Tris, 50 mM KCl, 4 mM MgCl_2_, 0.2 mM dNTPs, 0.5U Platinum *Taq* polymerase (Invitrogen, Carlsbad, CA), 700 nM each primer, and 250 nM probe. The assay was performed using a Rotor-Gene 3000 thermocycler (Corbett Life Sciences, Australia) using the following cycling conditions: 95°C for 5 min, followed by 40 cycles of 95°C for 10 sec and 60°C for 45 sec. DNA standards were made from total genomic DNA from *Y. pestis* and were diluted in nuclease-free water to yield a range of 10^6^ to 10^2^ genome copies per 2 µl. A standard curve using these dilutions was run in duplicate.

### Radiolabeled amino acid uptake

Actively growing *Y. pestis* (in BHI broth) and non-culturable cells from the autoclaved tap-water microcosm were used in the radiolabeled amino acid uptake experiments. For actively growing controls, 10 µl of *Y. pestis* cultured statically for 48 hours at 37°C in BHI broth were washed twice in PBS and resuspended in 5 ml of fresh BHI broth containing 1 µCi/ml of ^35^S-labeled cysteine/methionine amino acid mix. ^35^S-labeled cysteine and methionine were also added (1 µCi/ml final contraction) to an aliquot of non-culturable cells taken from the tap water microcosm. All cultures were incubated in the dark. Aliquots were removed at the time-points indicated in figure 3 for analysis. At selected time points, a 0.5 ml sample of either actively growing or non-culturable *Y. pestis* was removed, washed three times with PBS, and fixed with 2% (v/v) paraformaldehyde. The fixed samples were pelleted by centrifugation at 14,000×g for 30 min, and the cell pellet was resuspended in Laemmli sample loading buffer. The samples were boiled at 100°C for 5 min and electrophoresed through an 8% SDS-PAGE gel. The gel was dried and exposed to X-ray film for 48 h at −80°C. Quantification of each lane was performed using the spot densitometry function in the AlphaEaseFC software package by Alpha Innotech.

### Scanning electron microscopy (SEM)

Actively growing (in BHI broth) or nonculturable *Y. pestis* cells from the tap water microcosms were harvested by centrifugation and the bacteria pellets were washed three times with sterile phosphate-buffered saline (PBS, pH 7.4). Cells were fixed in 4% (v/v) formaldehyde/1% (v/v) glutaraldehyde in Millonig buffer at room temperature for≥1 h. The samples were washed three times with PBS to remove fixative and dehydrated using a graded ethanol series (75%, 95%, and 100%) into propylene oxide. Samples were spotted onto filter paper, critically point dried in an Autosamdri-815 critical point dryer, and coated with palladium gold. The coated samples were analyzed in a Hitachi S-4500 field emission scanning electron microscope.

### Transmission electron microscopy (TEM)

Actively growing or nonculturable *Y. pestis* microcosms were harvested by centrifugation and the bacterial pellets were washed three times with PBS. Cells were fixed in 4% (v/v) formaldehyde/1% (v/v) glutaraldehyde in Millonig buffer at room temperature for at least ≥1 h. The samples were washed three times with PBS to remove fixative and post fixed in 1% osmium tetroxide in cacodylate buffer for 1 h, followed by washing in double-distilled water to remove osmium. Samples were dehydrated in three washes of 50% ethanol and stained in 0.5% uranyl acetate for 20 min. Samples were then dehydrated through a graded ethanol series (75%, 95%, and 100%) into propylene oxide. After dehydration, the samples were infiltrated with a 1∶1 ratio of epoxy:propylene oxide, and embedded in 100% EPON epoxy. Thin sections were cut using a Leica Ultracut UCT ultramicrotome, post stained with uranyl acetate and lead citrate; and examined under a JEOL 1011 transmission electron microscope.

### Resuscitation and most probable number test

Resuscitation experiments based on the ten tube most probable number test devised by Halvorson and Ziegler was used [26]. One milliliter of the nonculturable tap water microcosm was added to nine milliliters of media. Additionally, 10-fold dilution series of each microcosm were made. One milliliter of each dilution was added to nine milliliters of media. The media tested were 1) 12.5% Brain Heart Infusion (BHI) broth, 2) tap water, and 3) 2% Fetal Bovine Serum (FBS)(ATCC, Manassas, VA, USA). The cultures were incubated statically at room temperature and 37°C for 72 hours. The table provided in the original Halvorson and Ziegler manuscript was used to determine the most probable number of viable cells in each microcosm based on outgrowth at each particular dilution.

## Supporting Information

File S1 Excerpt of the 2007 Erie County Water Authority (ECWA) annual water quality report. This report provides information regarding water quality and constituents as determined by the ECWA during the time frame of experimentation.
(PDF)

File S2 Excerpt from the 2008 Erie County Water Authority (ECWA) annual water quality report. This report provides information regarding water quality and constituents as determined by the ECWA during the time frame of experimentation.
(PDF)
