# Supplementary material for: Entry of Yersinia pestis into the Viable but Nonculturable State in a Low-Temperature Tap Water Microcosm
Source: PLoS One. 2011 Mar 16;6(3):e17585. doi: 10.1371/journal.pone.0017585 (PMC3059211; doi:10.1371/journal.pone.0017585)
Supplement: File S1 — Excerpt of the 2007 Erie County Water Authority (ECWA) annual water quality report. This report provides information regarding water quality and constituents as determined by the ECWA during the time frame of experimentation. (PDF) [file pone.0017585.s001.pdf]

2007 System Improvements

In 2007, the ECWA invested over $19 million in system improvements. This included the replacement of Gartman and Chestnut Ridge pumping stations, installation of more efficient, variable speed pumps at treatment plants and pumping stations, placement of additional standby power generators at main production and pumping facilities, security upgrades and waterline improvements. Waterlines were replaced in the towns of Amherst, Cheektowaga, Hamburg and West Seneca, the Village of Depew, and the Cities of Lackawanna and Tonawanda. The ECWA also initiated a change to new, radio read water meters which will offer convenience to customers as well as ensure more accurate billing.

The ECWA will continue to maintain its aggressive system wide improvement program with an additional $29.5 million capital spending plan included in the 2008 budget.

Questions?

If you would like additional copies of this report, please contact the Public Affairs Office at (716)849-8406 or email to dnemoyer@ecwa.org.

Thank you for allowing the ECWA to continue to provide you with quality drinking water. The ECWA is committed to providing you with information about your water supply. Customers who are well informed are our best allies in supporting improvements necessary to maintain the highest drinking water standards.

Any member of the public may participate in decisions affecting the quality of water. The ECWA's Board of Commissioners ultimately makes those decisions on behalf of our customers. Board meetings take place every other Thursday at 4:00 p.m. in the board meeting room, Erie County Water Authority, 350 Ellicott Square Building, 295 Main Street, Buffalo, New York 14203. Occasionally a board meeting is rescheduled. Call (716)849-8484 or visit www.ecwa.org for updated board meeting information.

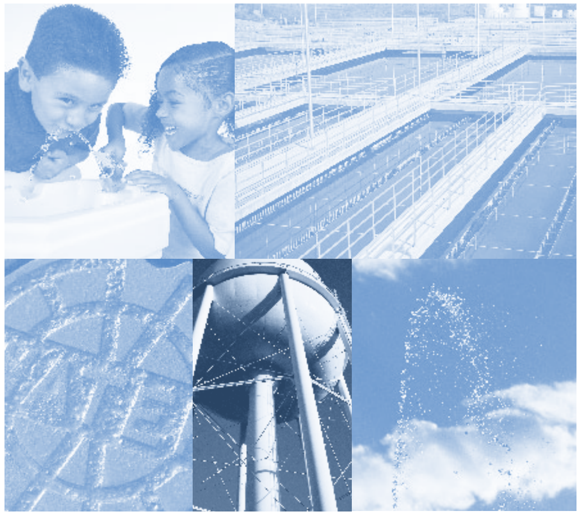

PUBLIC WATER SYSTEMS IDENTIFICATION NUMBERS
ECWA PWS# NY 1400443

PWS#	Name	PWS#	Name
NY1400397	AKRON VILLAGE	NY1400495	CLOVER BANK WD
NY1400398	ALDEN VILLAGE	NY1400496	EAST FRONTIER DRIVE WD
NY1400399	AMHERST WD#1	NY1400497	GLENDALE HEIGHT WD
NY1400400	AMHERST WD#2	NY1400498	HOLLYWOOD WATER DISTRICT
NY1400401	AMHERST WD#3	NY1400499	LAKESHORE WD
NY1400402	AMHERST WD#4	NY1400500	LYTH WD
NY1400403	AMHERST WD#5	NY1400501	MOUNT VERNON WD
NY1400404	AMHERST WD#6	NY1400502	SALEM DR WD
NY1400405	AMHERST WD#7	NY1400503	WINDOVER WATER DISTRICT
NY1400406	AMHERST WD#8	NY1400504	WOODLAWN WD
NY1400407	AMHERST WD#9	NY1400506	LACKWANNA CITY
NY1400408	AMHERST WD#10	NY1400508	LANCASTER WD#1
NY1400409	AMHERST WD#11	NY1400509	LANCASTER WD#2
NY1400410	AMHERST WD#12	NY1400510	LANCASTER WD#3
NY1400411	ANGOLA VILLAGE	NY1400511	LANCASTER WD#4
NY1400412	AURORA WD#1	NY1400512	LANCASTER WD#5
NY1400415	AURORA WD#4	NY1400513	LANCASTER WD#6
NY1400417	AURORA WD#6	NY1400514	LANCASTER WD#7
NY1400418	AURORA WD#7	NY1400515	LANCASTER WD#8
NY1400419	AURORA WD#8	NY1400518	ORCHARD PARK WD#1
NY1400421	BOWMANSVILLE WD	NY1400519	ORCHARD PARK WD#2
NY1400424	BELLVUE WD	NY1400520	WEBSTERS CORNER WD
NY1400425	CHEEKTOWAGA WD#9	NY1400521	WINDHAM ABBOTT ROAD WD
NY1400426	DOYLE WD	NY1400523	ORCHARD PARK WD#4
NY1400427	CHEEKTOWAGA WD#10	NY1400524	ORCHARD PARK WD#5
NY1400428	CHEEKTOWAGA WD#8	NY1400525	ORCHARD PARK WD#6
NY1400289	CHEEKTOWAGA WD#11	NY1400526	ORCHARD PARK WD#7
NY1400432	DEPEW VILLAGE	NY1400527	ORCHARD PARK WD#8
NY1400434	EAST HAMBURG WD#1	NY1400528	ORCHARD PARK WD#9
NY1400435	EDEN WD#1	NY1400529	ORCHARD PARK WD#10
NY1400436	EDEN WD#2	NY1400530	ORCHARD PARK WD#11
NY1400437	EDEN WD#3	NY1400531	ORCHARD PARK WD#12
NY1400438	EDEN WD#4	NY1400532	ORCHARD PARK WD#13
NY1400439	EDEN WD#5	NY1400533	ORCHARD PARK WD#15
NY1400440	EDEN WD#6	NY1400534	ORCHARD PARK WD#17
NY1400441	EDEN WD#7	NY1400535	ORCHARD PARK WD#19
NY1400442	EDEN WD#8	NY1404543	WEST SENECA WD NO1
NY1400445	EVANS WD#2	NY1404544	WEST SENECA WD NO2
NY1400446	EVANS WD#3	NY1404545	WEST SENECA WD NO3
NY1400447	EVANS, TOWN WATER DEP.	NY1404546	WEST SENECA WD NO4
NY1400448	FARNHAM VILLAGE	NY1404547	WEST SENECA WD NO5
NY1400462	ABBOTT HIGHLAND WD	NY1404548	WEST SENECA WD NO6
NY1400463	BURKE WD	NY1404549	WEST SENECA WD NO7
NY1400464	CENTRAL HAMBURG WD	NY1404550	WEST SENECA WD NO8
NY1400465	CHESTNUT RIDGE WATER	NY1404551	WEST SENECA WD NO9
NY1400466	HAMBURG WD#1	NY1404562	MEADOWBROOK WD#12
NY1400467	HAMBURG WD#2	NY1404566	CLEVELAND HILL WD
NY1400468	BAYVIEW ROAD WD	NY1410128	ORCHARD PARK WD#3
NY1400469	BEACON HILL WD	NY1410142	KENMORE VILLAGE
NY1400470	BEETOW DRIVE WD	NY1419099	ORCHARD PARK WD #18
NY1400471	BONNIE LANE WD	NY1419527	EVANS WD#4
NY1400472	HAMBURG ORCHARD PARK	NY1419528	EVANS WD#5
NY1400473	KNOB LILLYDALE BENZ WD	NY1420549	ELMA WATER DISTRICT
NY1400474	LAKEVIEW WD	NY1420550	AURORA WD#1A
NY1400475	LEWIS DRIVE WD	NY1420551	AURORA WD#9
NY1400476	MEADOWBROOK GREENFIELD	NY1420767	CLARENCE, TOWN WATER
NY1400477	OCKLER CAMP ROAD WD	NY1421651	ALDEN WD#1
NY1400478	OLD LAKEVIEW ROAD WD	NY1421652	ALDEN WD#2
NY1400479	MCKINLEY WD#1	NY1421653	ALDEN WD#3
NY1400480	OSBORNE SAGAMORE HEIGHTS	NY1421761	ORCHARD PARK WD#14
NY1400481	PARKER BIG TREE ROAD WD	NY1421897	BOSTON WD#1
NY1400482	PICTURE LAKE WD	NY1421898	BOSTON WD#2
NY1400483	SHORE HEIGHTS WD	NY1422651	NEWSTEAD WD#1
NY1400484	SOUTH TOWN WATER DIST	NY1422652	NEWSTEAD WD#2
NY1400485	STALEY DRIVE WD	NY1422653	NEWSTEAD WD#3
NY1400486	THRUWAY WD	NY1422654	NEWSTEAD WD#4
NY1400487	VAIL WD	NY1430016	NEWSTEAD #8
NY1400488	ATHOL SRINGS LOCKSLEY	NY1443000	NEWSTEAD WD#6
NY1400489	BAIN WD	NY1404557	TONAWANDA CON. WATER
NY1400490	BETHFORD LAKE WD	NY1400538	CITY OF TONAWANDA
NY1400491	BIG TREE GARDEN WD	NY1450020	COLDEN WD#1
NY1400492	BRISTOL WD	NY1450018	NEWSTEAD WD#10
NY1400493	CAMP ROAD LAKESHORE WD	NY6030016	BENNINGTON
NY1400494	CLARK STREET WD		

2007 Water Quality Report

Erie County Water Authority
Administrative Offices
295 Main St. Suite 350
Buffalo, NY 14203
716/849-8484 • www.ecwa.org

PRSRT STD
US POSTAGE PAID
HAMBURG, NY
PERMIT NO. 159

Dear Customer,

For 2007, your tap water met all State drinking water health standards. We are proud to report that our system did not violate a maximum contaminant level or any other water quality standard.

The Erie County Water Authority (ECWA) is committed to providing its customers safe, high quality drinking water. That is why the ECWA maintains a rigorous quality control program and continues to invest substantial financial resources to improve our two treatment facilities, distribution system and nationally recognized water quality lab. Our water is constantly monitored and tested. Each year ECWA strives to provide its customers with the high quality drinking water that they deserve.

As we enter a new year, the ECWA has positioned itself to continue to achieve its mission of providing a high-quality product and reliable, cost-effective service at an affordable rate to the more than 550,000 consumers that rely on us every day, 24 hours a day, 365 days a year.

Therefore, it is with pleasure that we provide you with the ECWA's 2007 Annual Water Quality Report (AWQR). This report provides an overview of the ECWA's water quality during the past year. It shows the source of your water, how it compares to standards set by regulatory agencies, how your water is treated and tested, discusses ECWA programs to improve your water quality and answers common questions asked by our customers. This report fulfills the United States Environmental Protection Agency's requirement to prepare and deliver a Consumer Confidence Report (CCR) and the New York State Department of Health's requirement to prepare and deliver an Annual Water Quality Report (AWQR).

The ECWA's highly trained staff looks forward to continuing to bring our most abundant, our most precious, our most natural resource into the homes, the businesses and the lives of the residents of Western New York. Your comments and questions about this report are important to us. Please forward them to:
Dan NeMoyer, Public Affairs Officer, 295 Main Street, Suite 350, Buffalo, N.Y. 14203, phone 849-8406, or email to dnemoyer@ecwa.org.

Sincerely,

BOARD OF COMMISSIONERS
Frank E. Swiatek, Chairman
Kelly M. Vacco, Vice-Chair
Francis G. Warthling, Treasurer

Where Does My Water Come From?

In general, the sources of drinking water (both tap water and bottled water) include rivers, lakes, streams, ponds, reservoirs, springs, and wells. As water travels over the surface of the land or through the ground, it dissolves naturally occurring minerals and, in some cases, radioactive material, and can pick up substances resulting from the presence of animals or from human activities. Contaminants that may be present in source water include: microbial contaminants; inorganic contaminants; pesticides and herbicides; organic chemical contaminants; and radioactive contaminants. In order to ensure that tap water is safe to drink, the State and the EPA prescribe regulations which limit the amount of certain contaminants in water provided by public water systems. The State Health Department's and the FDA's regulations establish limits for contaminants in bottled water which must provide the same protection for public health.

Your water comes from two sources. The ECWA's Sturgeon Point Treatment Plant in the Town of Evans draws water from Lake Erie to supply the southern part of Erie County and some communities in Chautauqua and Cattaraugus County. The Van de Water Treatment Plant in Tonawanda draws water from the "mighty" Niagara River and services municipalities in northern Erie County as well as some in Genesee County and Wyoming County. These two plants serve more than a half million people in Western New York.

What is the Erie County Water Authority?

The ECWA was created in 1949 by a special act of the New York State Legislature to ensure that the people and industry of Erie County would have a safe, plentiful supply of water for the future.

Since 1953, the ECWA has produced and reliably delivered to its customers water of the highest quality at an affordable rate.

As an independent public-benefit corporation, the ECWA is a financially self-sustaining business enterprise, and pays all operating expenses from revenues generated by the sale of water to its 157,163 customers. The ECWA is not an agency of New York State and is totally independent of Erie County government.

In 2007 the ECWA produced roughly 27 billion gallons of high-quality water for residential, commercial, and industrial use in 34 municipalities throughout Western New York. Some of this water was used for flushing water mains, fighting fires, training firefighters, filter backwashing and plant processes, equipment and hydrant testing and some of this water was lost to leaks. Approximately 19.5 billion gallons were sold to our customer.

The ECWA owns and operates two water treatment plants, a nationally recognized water quality lab, 38 pump stations, 40 water storage tanks and maintains 3,372 miles of water mains, 17,126 fire hydrants, 31,039 valves and numerous appurtenances.

The ECWA's current residential rate of $2.86 per 1,000 gallons of delivered water is one of the lowest in New York State.

Service Area Map

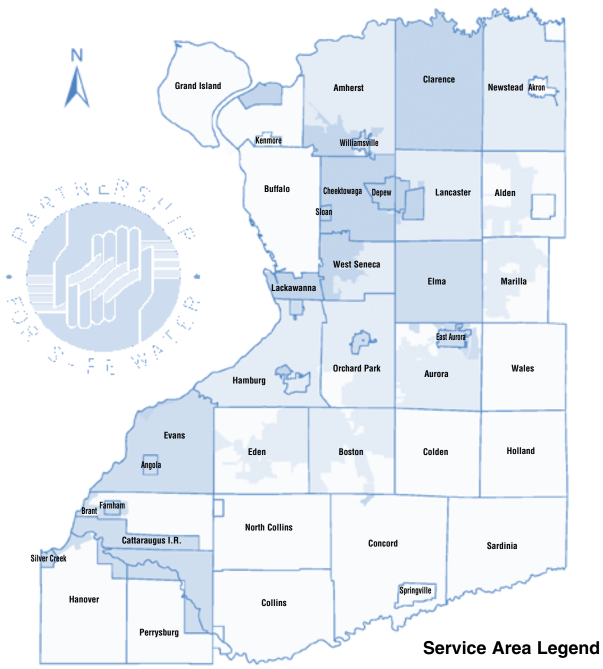

Who Sets and Enforces Drinking Water Standards?

The Safe Drinking Water Act (SDWA) is the main federal law that ensures the quality of your drinking water. Under the SDWA, the United States Environmental Protection Agency (EPA) sets standards for drinking water quality and oversees the states, localities, and water suppliers who implement those standards. In New York, the State Health Department enforces the EPA's regulations and often makes them even more stringent.

The EPA sets standards for approximately 150 regulated contaminants in drinking water. For each of these contaminants, EPA sets a legal limit, called a maximum contaminant level (MCL). EPA regulations specify strict testing and reporting requirements for each contaminant. Water suppliers may not provide water that doesn't meet these standards. Water that does meet these standards is safe to drink. In Erie County, the Erie County Health Department is the agency that administers and enforces these standards. Their phone number is (716)858-6964.

Drinking water, including bottled water, may reasonably be expected to contain at least small amounts of some contaminants. The presence of contaminants does not necessarily indicate that the water poses a health risk. More information about contaminants and potential health effects can be obtained by calling the Safe Drinking Water Hotline at (800)426-4791.

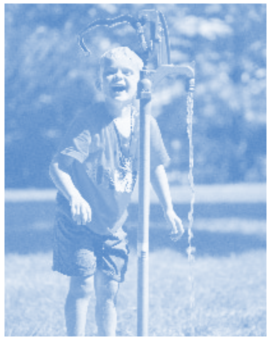

How Is My Water Treated?

The ECWA's two water treatment facilities use the conventional filtration method. At the plants, water undergoes the following treatment steps:

- Raw water flows by gravity through a large intake tunnel to the raw water building.
- Pumps draw the water through traveling screens to prevent large objects such as driftwood and fish from entering the system.
- A chemical, polyaluminum chloride, is added to the water, which causes suspended particles in the water to clump together to form floc.
- Floc particles then settle to the bottom of large sedimentation basins.
- The water is filtered through layers of anthracite, sand, and gravel, to remove any remaining particles.
- Chlorine is added for disinfection to kill bacteria. Small amounts of fluoride are added to help prevent tooth decay.
- Caustic soda is added to stabilize the alkalinity of the water and prevent corrosion in home plumbing.
- Powdered activated carbon is added in summer months to help remove unpleasant tastes and odors.
- Water is temporarily stored in clearwells or storage tanks before it is pumped to the public.
- High service pumps deliver the clean water through more than 3,372 miles of pipeline to homes and businesses. The ECWA closely monitors its 38 pump stations and 40 water storage tanks to assist in the distribution process. On average, the ECWA delivers 68 million gallons a day to customers.

Are there contaminants in our water? Do I need to take special precautions?

It should be noted that all drinking water, including bottled drinking water, my be reasonably expected to contain at least small amounts of some contaminants. The presence of contaminants does not necessarily indicate that water poses a health risk. More information about contaminants and potential health effects can be obtained by calling the EPA's Safe Drinking Water Hotline (800-426-4791) or the Erie County Health Department at 858-6964.

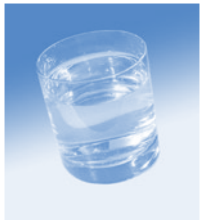

Although our drinking water met or exceeded state and federal regulations, some people may be more vulnerable to disease causing microorganisms or pathogens in drinking water than the general population. Immuno-compromised persons such as persons with cancer undergoing chemotherapy, persons who have undergone organ transplants, people with HIV/AIDS or other immune system disorders, some elderly, and infants can be particularly at risk from infections. These people should seek advice from their health care provider about their drinking water.

EPA/CDC guidelines on appropriate means to lessen the risk of infection by Cryptosporidium, Giardia and other microbial pathogens are available from the Safe Drinking Water Hotline: 800-426-4791.

How Is My Water Tested and Who Is Responsible for Making Sure It's Safe?

The ECWA conducts more than 70,000 tests annually to make sure all federal and state drinking water regulations are met. Our water is tested 24 hours a day, 365 days a year to assure the delivery of safe, clean water to every customer's tap. The ECWA operates three New York State-certified laboratories, one located at each

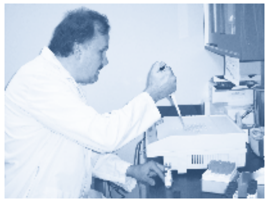

water treatment plant and a nationally recognized water quality laboratory in Lackawanna, which contains state-of-the-art testing equipment. The National Environmental Laboratory Accreditation Program (NELAP) certifies each of these laboratories. NELAP is a national accrediting body, made up of state, federal and commercial laboratory accreditation officials, that sets strict standards for public and commercial laboratories across the country.

Highly trained water treatment plant operators perform hourly tests at each phase of the treatment process. Our professional water quality staff also collects over 200 samples a month from the distribution system and tests for organic and inorganic compounds, and microbial contaminants. The results are sent to both the New York State and Erie County Health Departments to confirm that the ECWA is meeting all of its regulatory requirements.

The ECWA employs 254 dedicated professionals who continuously participate in educational training, licensing programs and professional associations to develop their skills to the highest possible levels.

These people live in your communities, are your friends and drink the same water you do. No wonder they are committed to making sure that your water is pure, safe and affordable.

Conservation Tips

Except for the air we breathe, water is the single most important element in our lives. It's too precious to waste. In an effort to make the most efficient use of our water resources, the ECWA encourages customers to practice the following water conservation measures to preserve our most precious resource:

- Use the clothes washer for full loads only.
- Instead of letting the water run in the sink when you want a cold drink, keep a jug or pitcher in the refrigerator.
- Turn the water off while you brush your teeth.
- Take shorter showers. A shower uses about 10 gallons a minute. Time yourself.
- Check your toilet for leaks by putting a few drops of food coloring in your tank. If the color shows up in your toilet bowl without flushing, you have a leak that is costing you money and wasting water.
- Check every faucet in your home for leaks. Just a slow drip can waste 20 gallons a day.
- Sweep outside with a broom, not a hose.
- Only water your lawn when necessary. If the grass springs back after you step on it, then it does not need to be watered.

Is the Public Informed If the Water Is Not Safe to Drink?

EPA regulations mandate the ECWA notify its customers if water is not safe to drink. Water is not safe to drink when testing reveals that contaminants in the water exceed national limits for contaminant levels. In the unlikely event that water becomes unsafe to drink, the ECWA will issue a "boil water order" and notify the public by newspaper, television and radio announcements.

Cryptosporidium and *Giardia* Analysis?

The ECWA's Water Quality Laboratory is recognized as one of the most well equipped labs in North America that is capable of testing for Giardia and Cryptosporidium. In fact, our lab was one of the first labs in the country to gain EPA approval for the analysis of Cryptosporidium and Giardia, and continues to participate in the EPA's Laboratory Quality Assurance Evaluation Program for the analysis of Cryptosporidium. The ECWA also tests for these protozoa for other major public water suppliers throughout the country.

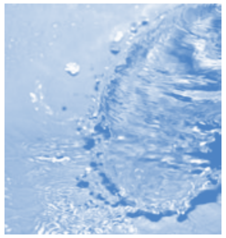

These microscopic protozoa are widely present in the environment and most surface water sources throughout the United States. They can cause intestinal illnesses if ingested. Symptoms of infection include nausea, diarrhea and abdominal cramps. Most healthy individuals can overcome the illnesses within a couple of weeks. However, both can be serious for people with weak immune systems such as those undergoing chemotherapy, dialysis or transplant patients and people with Crohn's disease or HIV infection.

In 2007, the ECWA analyzed 48 water samples for Giardia and Cryptosporidium. No positive samples were detected in the ECWA's treated water supply. Giardia and Cryptosporidium were found to be present in our source water. Specific test results are listed in the table below.

The ECWA encourages immune compromised individuals to consult their physicians regarding appropriate precautions to avoid infection. Both protozoa must be ingested to cause disease, and they may spread through other means than drinking water. For additional information on Cryptosporidiosis or Giardiasis, please contact the Erie County Health Department at (716) 858-6964.

ECWA's Test Results for 2007

The ECWA's water system operated under "NO VARIANCE OR EXEMPTION" from any federal or state regulatory requirements.

To comply with EPA mandated requirements, water quality data tables of detected regulated and unregulated contaminants are detailed in this report. The tables summarize test results for the past year or from the most recent year that tests were conducted in accordance with regulatory requirements. They also list the maximum contaminant levels (MCL). The EPA is responsible for establishing the MCL standards. For your convenience, important terms and abbreviations are defined throughout this document.

More information regarding all substances tested for, but not detected, can be obtained by calling the Customer Service Department at 849-8484.

Erie County Water Authority - Providing Water You Can Trust!

ERIE COUNTY WATER AUTHORITY • PWSID # 1400443

2007 Water Quality Monitoring Report – Annual Water Quality Report Supplement

DETECTED CONTAMINANTS

Metals, Inorganics, Physical Tests	Violation Yes/No	Sample Date (or date of highest detected)	MCL	MCLG	Level Detected	Sources in Drinking Water
Arsenic	No	12/07	10 ug/liter	NE	0.62 - 0.72 ug/liter, Average = 0.67	Erosion of natural deposits; orchard runoff, glass and electronic production waste
Asbestos	No	8/07	7 MFL	7 MFL	ND - 0.2 MFL, Average = ND	Erosion of natural deposits; decay of asbestos cement water mains
Barium	No	10/03	2 mg/liter	NE	0.021 mg/liter	Erosion of natural deposits; drilling and metal wastes
Chloride	No	12/07	250 mg/liter	NE	18 - 26 mg/liter ; Average = 19	Naturally occurring in source water
Chlorine	No	7/07	MRDL = 4.0 mg/liter	MRDLG = 4 mg/liter	<0.20 to 2.2 mg/liter; Average = 0.76	Added for disinfection
Fluoride	No	4/07	2.2 mg/liter	2.2 mg/liter	0.04 -1.27 mg/liter; Average = 0.77	Added to water to prevent tooth decay
Lead[1]	No	9/07	15 ug/liter (AL)	0 ug/liter (AL)	ND-38 ug/liter, 90th percentile 4 ug/liter, 1 of 97 was above AL	Home plumbing corrosion; natural erosion
Nitrate	No	12/07	10 mg/liter	10 mg/liter	0.21 to 0.24 mg/liter ; Average = 0.22	Runoff from fertilizer use
pH	No	7/07	NR	NE	6.8-8.8 SU; Average = 8.0	Naturally occurring; adjusted for corrosion control
Turbidity[2]	No	8/07	TT	NE	0.42 NTU highest detected; 97.3% was lowest monthly % < 0.30 NTU	Soil runoff

1 Lead is not present in the drinking water that is treated and delivered to your home. Lead in drinking water is primarily from materials and components associated with service lines and home plumbing. If present, elevated levels of lead can cause serious health problems, especially for pregnant women and young children. The Erie County Water Authority is responsible for providing high quality drinking water, but cannot control the variety of materials used in plumbing components. When your water has been sitting for several hours, you can minimize the potential for lead exposure by flushing your tap for 30 seconds to 2 minutes before using water for drinking or cooking. If you are concerned about lead in your water, you may wish to have your water tested. Information on lead in drinking water, testing methods, and steps you can take to minimize exposure is available from the Safe Drinking Water Hotline (800-426-4791) or at http://www.epa.gov/safewater/lead.

2 Turbidity is a measure of the cloudiness of water. ECWA monitors turbidity because it is a good indicator of the effectiveness of our filtration system. Turbidity has no health effects. However, turbidity can interfere with disinfection and provide a medium for bacterial growth. Turbidity may indicate the presence of disease-causing organisms. These organisms include bacteria, viruses, and parasites that can cause symptoms such as nausea, cramps, diarrhea, and associated headaches. On 8/09/07 the Van de Water Treatment Plant encountered a treatment upset which caused the combined effluent turbidity to exceed 0.3 ntu for a period of time. Corrective actions were taken throughout the day and into 8/10/07 until the plant turbidities were below the 0.3 ntu MCL. At no time did the plant readings exceed the maximum allowable treatment technique MCL. The combined filter turbidities were < 0.3 ntu 97.3% of the time for the month of August 2007.

Organic Compounds	Violation Yes/No	Sample Date (or date of highest detected)	MCL (ug/liter)	MCLG (ug/liter)	Level Detected (ug/liter)	Sources in Drinking Water
Total Trihalomethanes	No	8/07	RAA<80	NE	13-96 ug/liter ; RAA = 41.0	By-product of water disinfection (chlorination)
Total Haloacetic Acids	No	8/07	RAA<60	NE	5 - 54 ug/liter ; RAA = 19.9	By-product of water disinfection (chlorination)
Chloromethane[3]	No	12/07	5	NE	ND - 0.58 ug/liter; Average ND	Used in organic chemistry as an extractant and in industry as a solvent
1,2-Dichloroethane[3]	No	12/07	5	NE	ND - 0.61 ug/liter; Average ND	Discharge from industrial chemical factories
MIB and Geosmin	No	8/07	NR	NE	ND-4.5 ng/liter; Average < 2 (ND)	Taste and odor compounds from algae decomposition

3 Low levels of these compounds were detected in a sample taken 12/19/07 at the Sturgeon Point Treatment Plant. The low levels detected are not a violation of the MCL. Follow-up testing did not detect these compounds in the water. Some people who drink water containing 1,2-dichloroethane in excess of the MCL over many years may have an increased risk of getting cancer.

Radioactive Parameters	Violation Yes/No	Sample Date (or date of highest detected)	MCL	MCLG	Level Detected	Sources in Drinking Water
Gross Alpha	No	1/05	15.0 pCi/liter	0 pCi/liter	ND-1.7 pCi/liter	Erosion of natural deposits
Gross Beta	No	9/04	50** pCi/liter	0 pCi/liter	ND-2.2 pCi/liter	Decay of natural and man-made deposits
Combined Radium 226/ Radium 228	No	1/05	5.0 pCi/liter	0 pCi/liter	ND	Erosion of natural deposits
Total Uranium	No	6/04	30 ug/liter	0 ug/liter	ND-0.48 ug/liter	Erosion of natural deposits

*** New York State Department of Health considers 50 pCi/liter to be the level of concern for beta particles.*

Microbiological Parameters	Violation Yes/No	Sample Date (or date of highest detected)	MCL	MCLG	Level Detected	Sources in Drinking Water
Total Coliform Bacteria	No[4]	1/07	Any positive sample	0	0.47% = highest percentage of monthly positives	Naturally present in environment
E. coli Bacteria	No[5]	1/07	Any positive sample	0	2[6, 7]	Human and animal fecal waste

4 A violation occurs when more than 5% of the total coliform samples collected per month are positive.
5 A violation occurs when a total coliform positive sample is positive for E.coli and a repeat total coliform sample is positive or when a total coliform positive sample is negative for E.coli but a repeat total coliform sample is positive and the sample is also positive for E.coli.
6 On 1/16/07 a water sample taken at the Dodge Rd Elementary School was suspected of being positive for E.coli. Follow-up sampling and testing was performed and the results were negative for both total coliform & E.coli. No MCL violation occurred.
7 On 4/04/07 the Erie County Water Authority was issued a reporting violation for failing to report a suspected positive E.coli result within the required time frame. The organism was detected in a water sample taken 3/31/07 at the Van de Water Treatment Plant. Follow-up sampling and testing were performed and the results were negative for both total coliform & E.coli. No MCL violation occurred.

Giardia and Cryptosporidium	Violation Yes/No	Sample Date (or date of highest detected)	Number of Samples Testing Positive		Number of Samples Tested
			Giardia	Cryptosporidium	
Source Water	No	3/07	5	1	24
Treated Drinking Water	No	NA	0	0	24

Cryptosporidium is a microscopic pathogen found in surface waters throughout the United States, as a result of animal waste runoff. It can cause abdominal infection, diarrhea, nausea, and abdominal cramps if ingested. Our filtration process effectively removes Cryptosporidium. Cryptosporidium was not detected in any treated water samples taken in 2007. Giardia is a microbial pathogen present in varying concentrations in many surface waters. In 2007 Giardia was detected in 5 of 24 raw source water samples but was not detected in any treated drinking water samples. Giardia is removed/inactivated through a combination of filtration and disinfection or by disinfection alone.

UNREGULATED SUBSTANCES

Parameter	Sample Date (or date of highest detected)	MCL	MCLG	Average Level Detected (mg/liter)	Range (mg/liter)
Alkalinity	12/07	NR	NE	90	81-94
Calcium Hardness	1/07	NR	NE	90	65-99
Total Dissolved Solids	7/07	NR	NE	161	143-176
Total Organic Carbon	9/07	NR	NE	2.1	0.34-5.58

The seal of the Partnership for Safewater as seen on this document indicates that we are part of a select group of water systems nationwide who have voluntarily committed themselves toward a proactive approach to strengthen the safety of drinking water for our customers above and beyond the current regulatory requirements. For additional information on the Partnership for Safewater visit www.awwa.org/science/partnership.

ABBREVIATIONS AND TERMS

AL = Action Level: the concentration of a contaminant which, when exceeded, triggers treatment or other requirements which a water system must follow.

CFU/100 ml = Colony Forming Units per 100 milliliters

MCL= Maximum Contaminant Level: the highest level of a contaminant allowed in drinking water.

MCLG = Maximum Contaminant Level Goal: the level of a contaminant in drinking water below which there is no known or expected risk.

MFL = Million fibers/liter (Asbestos)

mg/liter = milligrams per liter (parts per million)

MRDL = Maximum Residual Disinfectant Level : the highest level of a disinfectant allowed in drinking water. There is convincing evidence that addition of a disinfectant is necessary for control of microbial contaminants.

MRDLG = Maximum Residual Disinfectant Level Goal: the level of a drinking water disinfectant below which there is no known or expected risk to health. MRDLGs do not reflect the benefits of the use of disinfectants to control microbial contamination

mrem/yr = millirems per year

ND = Not Detected: absent or present at less than testing method detection limit.

ng/liter = nanograms per liter = parts per trillion

NE = Not Established

NR = Not Regulated

NTU = Nephelometric Turbidity Units

pCi/liter = picocuries per liter

RAA = Running Annual Average

SU = Standard Units (pH measurement)

TT = Treatment Technique: a required process intended to reduce the level of a contaminant in drinking water.

ug/liter = micrograms per liter (parts per billion)

Variances and Exemptions= State or EPA permission not to meet an MCL or a treatment technique under certain conditions.

< : Denotes Less Than

≤ : Denotes Less Than or Equal To

Results are from 2007 analyses or from the most recent year that tests were conducted in accordance with regulatory requirements. Some tests are not required to be performed on an annual basis. Information can be obtained upon request from the ECWA Water Quality Laboratory (716) 685-8570 or on the Internet at www.ecwa.org.

As you can see by the tables, our system had no violations. We have learned through our testing that some contaminants have been detected; however, these contaminants were detected below the level allowed by the State.

COMPOUNDS OR ELEMENTS TESTED FOR BUT NOT DETECTED

2-Chlorotoluene	1,1,2-Trichloroethane	DCPA Monoacid degradate	Nitrite
4-Chlorotoluene	1,2,3-Trichloropropane	Dalapon	Nitrobenzene
2,4-D	1,1,2-Trichlorotrifluoroethane	Di(2-ethylhexyl) adipate	Oxamyl (Vydate)
4,4'-DDE	1,2,4-Trimethylbenzene	Di(2-ethylhexyl) phthalate	PCB 1016
DCPA monoacid degradate	1,3,5-Trimethylbenzene	Dibromomethane	PCB 1221
1,2-Dibromo-3-Chloropropane	Acetochlor	Dicamba	PCB 1232
DCPA monoacid degradate	Aldicarb	Dieldrin	PCB 1242
1,2-Dibromoethane	Aldicarb Sulfone	Dinoseb	PCB 1248
1,2-Dichlorobenzene	Aldicarb Sulfoxide	Diquat	PCB 1254
1,3-Dichlorobenzene	Aldrin	EPTC	PCB 1260
1,4-Dichlorobenzene	Antimony	Endothall	Pentachlorophenol
1,1-Dichloroethane	Atrazine	Endrin	Perchlorate
1,1-Dichloroethylene	Benzene	Ethylbenzene	Phosphate
cis-1,2-Dichloroethylene	Benzo(a)pyrene	Free Ammonia	Pichloram
trans-1,2-Dichloroethylene	Beryllium	Glyphosate	Propacchlor
1,2-Dichloropropane	Bromobenzene	Heptachlor	Propoxur
1,3-Dichloropropane	Bromochloromethane	Heptachlor Epoxide	n-Propylbenzene
2,2-Dichloropropane	Bromomethane	Hexachlorobenzene	Selenium
1,1-Dichloropropene	Butachlor	Hexachlorobutadiene	Silver
cis-1,3-Dichloropropene	n-Butylbenzene	Hexachlorocyclopentadiene	Simazine
trans-1,3-Dichloropropene	sec-Butylbenzene	Isopropylbenzene	Styrene
2,4-Dinitrotoluene	t-Butylbenzene	p-Isopropyltoluene	Terbacil
2,6-Dinitrotoluene	Cadmium	Lindane	Tetrachloroethylene
3-Hydroxycarbofuran	Carbaryl	Manganese	Thallium
1-Napthol	Carbofuran	Mercury	Toluene
2,3,7,8-TCDD (Dioxin)	Carbon Tetrachloride	Methiocarb	Toxaphene
2,4,5-TP (Silvex)	Chlordane	Methomyl	Trichloroethylene
1,1,1,2-Tetrachloroethane	Chlorobenzene	Methoxychlor	Trichlorofluoromethane
1,1,2,2-Tetrachloroethane	Chloroethane	Methyl t-butyl ether (MTBE)	Vinyl Chloride
1,2,3-Trichlorobenzene	Chromium	Methylene Chloride	Xylenes
1,2,4-Trichlorobenzene	Copper	Metolachlor	Zinc
1,1,1-Trichloroethane	Cyanide	Metribuzin	
1,1,2-Trichloroethane	DCPA Diacid degradate	Molinate	
Alachlor	Dichlorodifluoromethane	Napthalene	

New York State Department of Health Source Water Assessment

The New York State Department of Health completed a draft Source Water Assessment of the supply's raw water sources under the state's Source Water Assessment Program (SWAP). The purpose of this program is to compile, organize, and evaluate information regarding possible and actual threats to the quality of public water supply (PWS) sources. It is important to note that source water assessment reports estimate the potential for untreated drinking water sources to be impacted by contamination. These reports do not address the safety or quality of treated finished potable tap water. The Great Lakes' watershed is exceptionally large and too big for a detailed evaluation in the SWAP. General drinking water concerns for public water supplies, which use these sources include: storm generated turbidity, wastewater, toxic sediments, shipping related spills, and problems associated with exotic species (e.g. zebra mussels – intake clogging and taste and odor problems). The SWAP is based on the analysis of the contaminant inventory compiled for the drainage areas deemed most likely to impact drinking water quality at this public water supply's raw water intakes. Separate assessments were completed for the Lake Erie source and the Niagara River source. The assessment found a moderate susceptibility to contamination for the Lake Erie source. The amount of agricultural land in the assessment area results in elevated potential of disinfection byproduct precursors and pesticides contamination. While there are some facilities present, permitted discharges do not likely represent an important threat to source water quality based on their density in the assessment area. There is also noteworthy contamination susceptibility associated with other discrete contaminant sources, and these facility types include: landfills. The assessment found an elevated susceptibility to contamination for the Niagara River source. The amount of agricultural (and to a lesser extent residential) lands in the assessment area results in elevated potential for microbials, disinfection byproduct precursors, and pesticides contamination. There is also a high density of sanitary wastewater discharges, which results in elevated susceptibility for all contaminant categories. Non-sanitary wastewater discharges may also contribute to contamination. There is also considerable contamination susceptibility associated with other discrete contaminant sources, and these facility types include: chemical bulk storage, inactive hazardous waste sites, landfills, Resource Conservation and Recovery Act facilities and Toxics Release Inventory facilities.

If you have any questions about New York State's Source Water Assessment Program, please contact Ms. Dolores Funke, P.E., Senior Public Health Engineer, Erie County Health Department at 858-6966.
