# Supplementary material for: Entry of Yersinia pestis into the Viable but Nonculturable State in a Low-Temperature Tap Water Microcosm
Source: PLoS One. 2011 Mar 16;6(3):e17585. doi: 10.1371/journal.pone.0017585 (PMC3059211; doi:10.1371/journal.pone.0017585)
Supplement: File S2 — Excerpt from the 2008 Erie County Water Authority (ECWA) annual water quality report. This report provides information regarding water quality and constituents as determined by the ECWA during the time frame of experimentation. (PDF) [file pone.0017585.s002.pdf]

Conservation Tips

Except for the air we breathe, water is the single most important element in our lives. It's too precious to waste. In an effort to make the most efficient use of our water resources, the ECWA encourages customers to practice the following water conservation measures to preserve our most precious resource:

- Use the clothes washer for full loads only.
- Instead of letting the water run in the sink when you want a cold drink, keep a jug or pitcher in the refrigerator.
- Turn the water off while you brush your teeth.
- Take shorter showers. A shower uses about 10 gallons a minute. Time yourself.
- Check your toilet for leaks by putting a few drops of food coloring in your tank. If the color shows up in your toilet bowl without flushing, you have a leak that is costing you money and wasting water.
- Check every faucet in your home for leaks. Just a slow drip can waste 20 gallons a day.
- Sweep outside with a broom, not a hose.
- Only water your lawn when necessary. If the grass springs back after you step on it, then it does not need to be watered.

Questions?

If you would like additional copies of this report, please contact the Public Affairs Office at (716)849-8406 or email to dnemoyer@ecwa.org.

Thank you for allowing the ECWA to continue to provide you with quality drinking water. The ECWA is committed to providing you with information about your water supply. Customers who are well informed are our best allies in supporting improvements necessary to maintain the highest drinking water standards.

Any member of the public may participate in decisions affecting the quality of water. The ECWA's Board of Commissioners ultimately makes those decisions on behalf of our customers. Board meetings take place every other Thursday in the board meeting room, Erie County Water Authority, 350 Ellicott Square Building, 295 Main Street, Buffalo, New York 14203. Occasionally a board meeting is rescheduled. Call (716)849-8484 or visit www.ecwa.org for updated board meeting information.

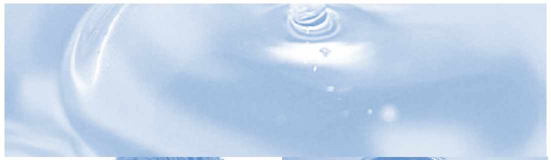

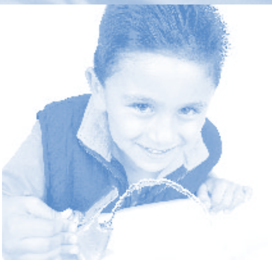

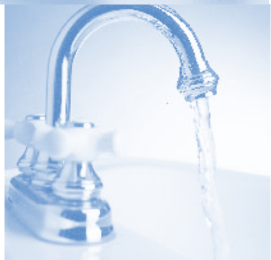

PUBLIC WATER SYSTEMS IDENTIFICATION NUMBERS
ECWA PWS# NY 1400443

PWS#	Name
NY1421651	ECWA ALDEN
NY1400399	ECWA AMHERST
NY1450033	ECWA AURORA
NY1421897	ECWA BOSTON
NY1400443	ECWA DIRECT
NY1400435	ECWA EDEN
NY1400488	ECWA HAMBURG (T)
NY1400515	ECWA HAMBURG (V)
NY1400421	ECWA LANCASTER
NY1430016	ECWA MARILLA
NY1422651	ECWA NEWSTEAD
NY1421762	ECWA ORCHARD PARK
NY1404543	ECWA WEST SENECA

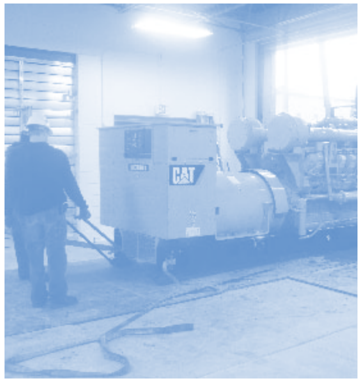

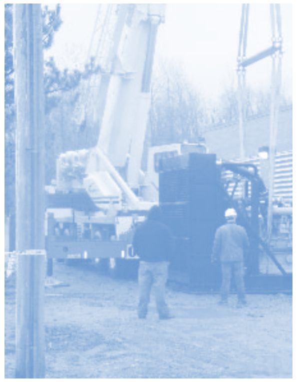

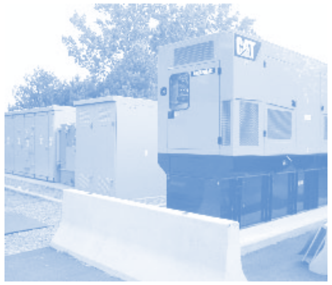

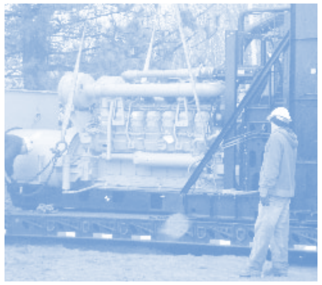

2008 System Improvements

In 2008, the ECWA invested over $33 million into Improvements to our water system. This included the ongoing reconstruction and improvements to the Sturgeon Point Water Treatment Plant settling basins and power system; the Installation of more efficient and effective variable drives at our Van De Water Treatment Plant and Ball Pumping Station; the replacement of numerous, large system valves; the purchase of additional standby power generators for our secondary and remote facilities, and waterline improvements. Waterline improvements were undertaken in the towns of Cheektowaga, Clarence, Lancaster, the Village of Depew, and the Cities of Lackawanna and Tonawanda. The ECWA also continues a change to new, radio read water meters which will offer convenience to customers as well as ensure more accurate billing.

The ECWA will continue to maintain its aggressive system wide improvement program with an additional $25 million capital spending plan included in the 2009 Budget.

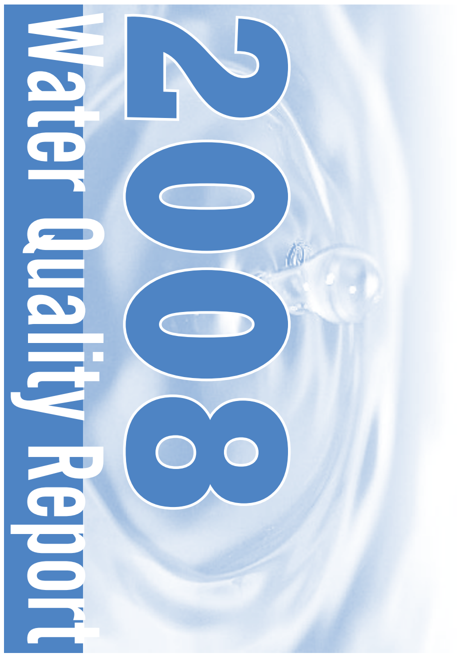

2008 Water Quality Report

ERIE COUNTY WATER AUTHORITY
Administrative Offices
295 Main St. Suite 350
Buffalo, NY 14203
716/849-8484 • www.ecwa.org

PRSRT STD
US POSTAGE PAID
HAMBURG, NY
PERMIT NO. 159

Dear Customer,

For 2008, your tap water met all State drinking water health standards. We are proud to report that our system did not violate a maximum contaminant level or any other water quality standard.

The Erie County Water Authority (ECWA) is committed to providing its customers safe, high quality drinking water. That is why the ECWA maintains a rigorous quality control program and continues to invest substantial financial resources to improve our two treatment facilities, distribution system and nationally recognized water quality lab. Our water is constantly monitored and tested. Each year ECWA strives to provide its customers with the high quality drinking water that they deserve.

As we enter a new year, the ECWA has positioned itself to continue to achieve its mission of providing a high-quality product and reliable, cost-effective service at an affordable rate to the more than 550,000 consumers that rely on us 24 hours a day, 365 days a year.

Therefore, it is with pleasure that we provide you with the ECWA's 2008 Annual Water Quality Report (AWQR). This report provides an overview of the ECWA's water quality during the past year. It shows the source of your water, how it compares to standards set by regulatory agencies, how your water is treated and tested, discusses ECWA programs to improve your water quality and answers common questions asked by our customers. This report fulfills the United States Environmental Protection Agency's requirement to prepare and deliver a Consumer Confidence Report (CCR) and the New York State Department of Health's requirement to prepare and deliver an Annual Water Quality Report (AWQR).

The ECWA's highly trained staff looks forward to continuing to bring our most precious, our most natural resource into the homes, the businesses and the lives of the residents of Western New York. Your comments and questions about this report are important to us. Please forward them to:
Dan NeMoyer, Director of Human Resources, ECWA, 295 Main Street, Room 350, Buffalo, N.Y. 14203, phone716-849-8406, or email to dnemoyer@ecwa.org.

Sincerely,

BOARD OF COMMISSIONERS
Frank E. Swiatek, Chairman
Kelly M. Vacco, Vice-Chair
Francis G. Warthling, Treasurer

Where Does My Water Come From?

In general, the sources of drinking water (both tap water and bottled water) include rivers, lakes, streams, ponds, reservoirs, springs, and wells. As water travels over the surface of the land or through the ground, it dissolves naturally occurring minerals and, in some cases, radioactive material, and can pick up substances resulting from the presence of animals or from human activities. Contaminants that may be present in source water include: microbial contaminants; inorganic contaminants; pesticides and herbicides; organic chemical contaminants; and radioactive contaminants. In order to ensure that tap water is safe to drink, the State and the EPA prescribe regulations which limit the amount of certain contaminants in water provided by public water systems. The State Health Department's and the FDA's regulations establish limits for contaminants in bottled water which must provide the same protection for public health.

Your water comes from two sources. The ECWA's Sturgeon Point Treatment Plant in the Town of Evans draws water from Lake Erie to supply the southern part of Erie County and some communities in Chautauqua and Cattaraugus County. The Van de Water Treatment Plant in Tonawanda draws water from the "mighty" Niagara River and services municipalities in northern Erie County as well as some in Genesee County and Wyoming County. These two plants serve more than a half million people in Western New York.

What is the Erie County Water Authority?

The ECWA was created in 1949 by a special act of the New York State Legislature to ensure that the people and industry of Erie County would have a safe, plentiful supply of water for the future.

Since 1953, the ECWA has produced and reliably delivered to its customers water of the highest quality at an affordable rate.

As an independent public-benefit corporation, the ECWA is a financially self-sustaining business enterprise, and pays all operating expenses from revenues generated by the sale of water to its 157,571 customers. The ECWA is not an agency of New York State and is totally independent of Erie County government.

In 2008 the ECWA produced just over 25 billion gallons of high-quality water for residential, commercial, and industrial use in 35 municipalities throughout Western New York. Some of this water was used for flushing water mains, fighting fires, training firefighters, filter backwashing and plant processes, equipment and hydrant testing and some of this water was lost to leaks. Approximately 17.5 billion gallons were sold to our customer.

The ECWA owns and operates two water treatment plants, a nationally recognized water quality lab, 38 pump stations, 40 water storage tanks and maintains 3,380 miles of water mains, 17,134 fire hydrants, over 30,000 valves and numerous appurtenances.

The ECWA's current residential rate of $2.86 per 1,000 gallons of delivered water is one of the lowest in New York State.

Service Area Map

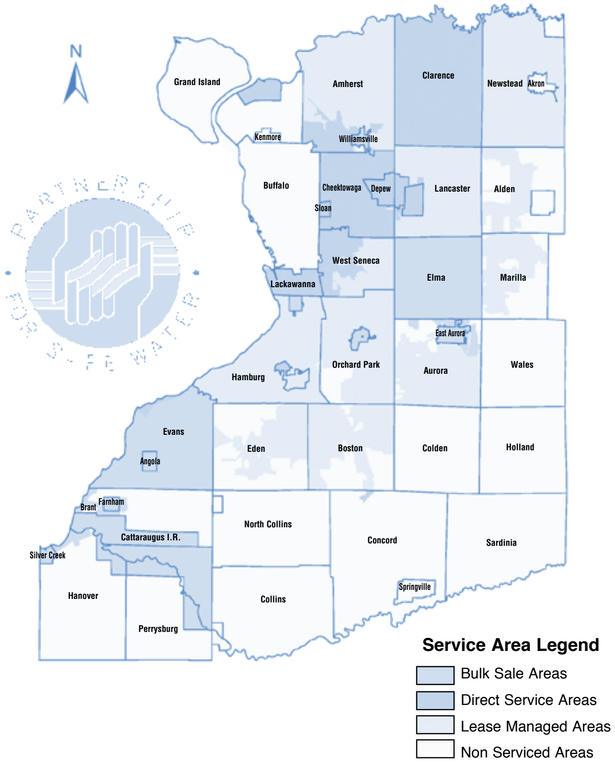

Who Sets and Enforces Drinking Water Standards?

The Safe Drinking Water Act (SDWA) is the main federal law that ensures the quality of your drinking water. Under the SDWA, the United States Environmental Protection Agency (EPA) sets standards for drinking water quality and oversees the states, localities, and water suppliers who implement those standards. In New York, the State Health Department enforces the EPA's regulations and often makes them even more stringent.

The EPA sets standards for approximately 150 regulated contaminants in drinking water. For each of these contaminants, EPA sets a legal limit, called a maximum contaminant level (MCL). EPA regulations specify strict testing and reporting requirements for each contaminant. Water suppliers may not provide water that doesn't meet these standards. Water that does meet these standards is safe to drink. In Erie County, the Erie County Health Department is the agency that administers and enforces these standards. Their phone number is (716)858-6089.

Drinking water, including bottled water, may reasonably be expected to contain at least small amounts of some contaminants. The presence of contaminants does not necessarily indicate that the water poses a health risk. More information about contaminants and potential health effects can be obtained by calling the Safe Drinking Water Hotline at (800)426-4791.

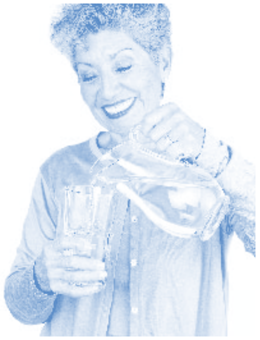

How Is My Water Treated?

The ECWA's two water treatment facilities use the conventional filtration method. At the plants, water undergoes the following treatment steps:

- Raw water flows by gravity through a large intake tunnel to the raw water building.
- Pumps draw the water through traveling screens to prevent large objects such as driftwood and fish from entering the system.
- A chemical, polyaluminum chloride, is added to the water, which causes suspended particles in the water to clump together to form floc.
- Floc particles then settle to the bottom of large sedimentation basins.
- The water is filtered through layers of anthracite, sand, and gravel, to remove any remaining particles.
- Chlorine is added for disinfection to kill bacteria. Small amounts of fluoride are added to help prevent tooth decay.
- Caustic soda is added to stabilize the alkalinity of the water and prevent corrosion in home plumbing.
- Powdered activated carbon is added in summer months to help remove unpleasant tastes and odors.
- Water is temporarily stored in clearwells or storage tanks before it is pumped to the public.
- High service pumps deliver the clean water through more than 3,380 miles of pipeline to homes and businesses. The ECWA closely monitors its 38 pump stations and 40 water storage tanks to assist in the distribution process. On average, the ECWA delivers 69 million gallons a day to customers.

Are there contaminants in our water? Do I need to take special precautions?

It should be noted that all drinking water, including bottled drinking water, my be reasonably expected to contain at least small amounts of some contaminants. The presence of contaminants does not necessarily indicate that water poses a health risk. More information about contaminants and potential health effects can be obtained by calling the EPA's Safe Drinking Water Hotline (800-426-4791) or the Erie County Health Department at 858-6089.

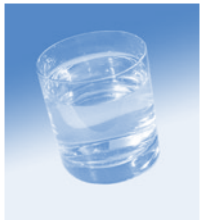

Although our drinking water met or exceeded state and federal regulations, some people may be more vulnerable to disease causing microorganisms or pathogens in drinking water than the general population. Immuno-compromised persons such as persons with cancer undergoing chemotherapy, persons who have undergone organ transplants, people with HIV/AIDS or other immune system disorders, some elderly, and infants can be particularly at risk from infections. These people should seek advice from their health care provider about their drinking water.

EPA/CDC guidelines on appropriate means to lessen the risk of infection by Cryptosporidium, Giardia and other microbial pathogens are available from the Safe Drinking Water Hotline: 800-426-4791.

How Is My Water Tested and Who Is Responsible for Making Sure It's Safe?

The ECWA conducts more than 70,000 tests annually to make sure all federal and state drinking water regulations are met. Our water is tested 24 hours a day, 365 days a year to assure the delivery of safe, clean water to every customer's tap. The ECWA operates three New York State-certified laboratories, one located at each water treatment plant and a nationally recognized water quality laboratory in Lackawanna, which contains state-of-the-art testing equipment. The National Environmental Laboratory Accreditation Program (NELAP) certifies each of these laboratories. NELAP is a national accrediting body, made up of state, federal and commercial laboratory accreditation officials, that sets strict standards for public and commercial laboratories across the country.

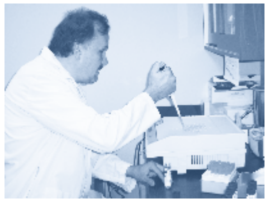

Highly trained water treatment plant operators perform hourly tests at each phase of the treatment process. Our professional water quality staff also collects over 220 samples a month from the distribution system and tests for organic and inorganic compounds, and microbial contaminants. The results are sent to both the New York State and Erie County Health Departments to confirm that the ECWA is meeting all of its regulatory requirements.

The ECWA employs 254 dedicated professionals who continuously participate in educational training, licensing programs and professional associations to develop their skills to the highest possible levels.

These people live in your communities, are your friends and drink the same water you do. No wonder they are committed to making sure that your water is pure, safe and affordable.

Fluoride

Our water system is one of the many drinking water systems in New York State that provides drinking water with a controlled, low level of fluoride for consumer dental health protection. According to the United States Centers for Disease Control, fluoride is very effective in preventing cavities when present in drinking water at an optimal range of 0.8 to 1.2 mg/l (parts per million). To ensure that the fluoride supplement in your water provides optimal dental protection, the New York State Department of Health requires that the Erie County Water Authority monitor fluoride levels on a daily basis. During the addition of fluoride in 2008, monitoring showed fluoride levels in your water were in the optimal range 100 % of the time. However, due to supply issues, the fluoride addition to your water was interrupted during the months of August, October and November. None of the monitoring results during fluoride addition showed fluoride at levels that approached the 2.2 mg/l MCL for fluoride.

Is the Public Informed If the Water Is Not Safe to Drink?

EPA regulations mandate the ECWA notify its customers if water is not safe to drink. Water is not safe to drink when testing reveals that contaminants in the water exceed national limits for contaminant levels. In the unlikely event that water becomes unsafe to drink, the ECWA will issue a "boil water order" and notify the public by newspaper, television and radio announcements.

Cryptosporidium and *Giardia* Analysis?

The ECWA's Water Quality Laboratory is recognized as one of the most well equipped labs in North America that is capable of testing for Giardia and Cryptosporidium. In fact, our lab was one of the first labs in the country to gain EPA approval for the analysis of Cryptosporidium and Giardia, and continues to participate in the EPA's Laboratory Quality Assurance Evaluation Program for the analysis of Cryptosporidium. The ECWA also tests for these protozoa for other major public water suppliers throughout the country.

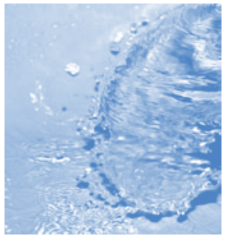

These microscopic protozoa are widely present in the environment and most surface water sources throughout the United States. They can cause intestinal illnesses if ingested. Symptoms of infection include nausea, diarrhea and abdominal cramps. Most healthy individuals can overcome the illnesses within a couple of weeks. However, both can be serious for people with weak immune systems such as those undergoing chemotherapy, dialysis or transplant patients and people with Crohn's disease or HIV infection.

In 2008, the ECWA analyzed 42 water samples for Giardia and Cryptosporidium. No positive samples were detected in the ECWA's treated water supply. Giardia were found to be present in our source water. Specific test results are listed in the table below.

The ECWA encourages immune compromised individuals to consult their physicians regarding appropriate precautions to avoid infection. Both protozoa must be ingested to cause disease, and they may spread through other means than drinking water. For additional information on Cryptosporidiosis or Giardiasis, please contact the Erie County Health Department at (716) 858-6089.

ECWA's Test Results for 2008

The ECWA's water system operated under "NO VARIANCE OR EXEMPTION" from any federal or state regulatory requirements.

To comply with EPA mandated requirements, water quality data tables of detected regulated and unregulated contaminants are detailed in this report. The tables summarize test results for the past year or from the most recent year that tests were conducted in accordance with regulatory requirements. They also list the maximum contaminant levels (MCL). The EPA is responsible for establishing the MCL standards. For your convenience, important terms and abbreviations are defined throughout this document.

More information regarding all substances tested for, but not detected, can be obtained by calling the Customer Service Department at 849-8484.

Erie County Water Authority - Providing Water You Can Trust!

ERIE COUNTY WATER AUTHORITY • PWSID # 1400443

2008 Water Quality Monitoring Report – Annual Water Quality Report Supplement

DETECTED CONTAMINANTS

Metals, Inorganics, Physical Tests	Violation Yes/No	Sample Date (or date of highest detected)	MCL	MCLG	Level Detected	Sources in Drinking Water
Arsenic	No	10/08	10 ug/liter	NE	0.71 - 0.78 ug/liter, Average = 0.74	Erosion of natural deposits; orchard runoff, glass, electronic production waste
Asbestos	No	8/07	7 MFL	7 MFL	ND - 0.2 MFL, Average = ND	Erosion of natural deposits; decay of asbestos cement water mains
Chloride	No	2/08	250 mg/liter	NE	17 - 39 mg/liter , Average = 21	Naturally occurring in source water
Chlorine	No	9/08	MRDL = 4.0 mg/liter	MRDLG = 4 mg/liter	<0.20 to 2.2 mg/liter, Average = 0.77	Added for disinfection
Fluoride[1]	No	5/08	2.2 mg/liter	2.2 mg/liter	0.11 - 1.71 mg/liter, Average = 0.97	Added to water to prevent tooth decay
Lead[2]	No	9/07	15 ug/liter (AL)	0 ug/liter (AL)	ND - 38 ug/liter, 90th percentile 4 ug/liter, 1 of 97 above AL	Home plumbing corrosion; natural erosion
Nitrate	No	10/08	10 mg/liter	10 mg/liter	0.13 to 0.15 mg/liter, Average = 0.14	Runoff from fertilizer use
pH	No	10/08	NR	NE	6.4 - 8.7 SU, Average = 7.8	Naturally occurring; adjusted for corrosion control
Turbidity[3]	No	9/08	TT	NE	0.22 NTU highest detected; 100% was lowest monthly % < 0.3 NTU	Soil runoff

1 Our system is one of the many drinking water systems in New York State that provides drinking water with a controlled, low level of fluoride for consumer dental health protection. According to the United States Centers for Disease Control, fluoride is very effective in preventing cavities when present in drinking water at an optimal range from 0.8 to 1.2 mg/l (parts per million).To ensure that the fluoride supplement in your water provides optimal dental protection, the State Department of Health requires that the Erie County Water Authority monitor fluoride levels on a daily basis. During the addition of fluoride in 2008, monitoring showed fluoride levels in your water were in the optimal range 100 % of the time. However, due to supply issues, the fluoride addition to your water was interrupted during the months of August, October and November. None of the monitoring resultsduring fluoride addition showed fluoride at levels that approached the 2.2 mg/l MCL for fluoride.

2 Lead is not present in the drinking water that is treated and delivered to your home. Lead in drinking water is primarily from materials and components associated with service lines and home plumbing. If present, elevated levels of lead can cause serious health problems, especially for pregnant women and young children. The Erie County Water Authority is responsible for providing high quality drinking water, but cannot control the variety of materials used in plumbing components. When your water has been sitting for several hours, you can minimize the potential for lead exposure by flushing your tap for 30 seconds to 2 minutes before using water for drinking or cooking. If you are concerned about lead in your water, you may wish to have your water tested. Information on lead in drinking water, testing methods, and steps you can take to minimize exposure is available from the Safe Drinking Water Hotline (800-426-4791) or at http://www.epa.gov/safewater/lead. The level presented represents the 90th percentile of the 97 sites tested. A percentile is a value on a scale of 100 that indicates a percent of a distribution that is equal to or below it. The 90th percentile is equal to or greater than 90% of the lead or copper values detected in the water system. In this case, 97 samples were collected in the water system and the 90th percentile value for lead was the sixth highest value (4 ug/L). The action level for lead was exceeded at only one of the sites tested (38 ug/L). The action level for copper was not exceeded at any of the sites tested.

3 Turbidity is a measure of the cloudiness of water. ECWA monitors turbidity because it is a good indicator of the effectiveness of our filtration system. Turbidity has no health effects. However, turbidity can interfere with disinfection and provide a medium for bacterial growth. Turbidity may indicate the presence of disease-causing organisms. These organisms include bacteria, viruses, and parasites that can cause symptoms such as nausea, cramps, diarrhea, and associated headaches. Our highest single turbidity measurement (0.22 NTU) for the year occurred on 9/14/08. State regulations require that turbidity must always be below 1 NTU. The regulations require that 95% of the turbidity samples collected have measurements below 0.3 NTU. All measurements met the treatment technique for turbidity. The levels recorded were always within the acceptable range allowed and did not constitute a treatment technique violation.

Organic Compounds	Violation Yes/No	Sample Date (or date of highest detected)	MCL (ug/liter)	MCLG (ug/liter)	Level Detected (ug/liter)	Sources in Drinking Water
Total Trihalomethanes[4]	No	8/08	RAA <80	NE	19 - 85 ug/liter, RAA = 42 ug/liter	By-product of water disinfection (chlorination)
Total Haloacetic Acids[5]	No	8/08	RAA <60	NE	6 - 50 ug/liter, RAA = 19 ug/liter	By-product of water disinfection (chlorination)
MIB and Geosmin	No	9/08	NR	NE	ND - 4.5 ng/liter, Average < 2 (ND)	Taste and odor compounds from algae decomposition

4 Trihalomethanes are byproducts of the water disinfection process that occur when natural organic compounds react with the chlorine required to kill harmful organisms in the water. Some people who drink water containing trihalomethanes in excess of the MCL over many years may experience problems with their liver, kidneys, or central nervous system, and may have an increased risk of getting cancer. The level detected represents the highest running annual average of quarterly results. This result (42 ug/L) is below the MCL.

5 Haloacetic acids are byproducts of the water disinfection process required to kill harmful organisms. Some people who drink water containing haloacetic acids in excess of the MCL over many years may have an increased risk of getting cancer. The level detected represents the highest running annual average of quarterly results. This result (19 ug/L) is below the MCL.

Radioactive Parameters	Violation Yes/No	Sample Date (or date of highest detected)	MCL	MCLG	Level Detected	Sources in Drinking Water
Gross Alpha	No	1/05	15.0 pCi/liter	0 pCi/liter	ND - 1.7 pCi/liter	Erosion of natural deposits
Gross Beta	No	9/04	50** pCi/liter	0 pCi/liter	ND - 2.2 pCi/liter	Decay of natural and man-made deposits
Combined Radium 226/ Radium 228	No	1/05	5.0 pCi/liter	0 pCi/liter	ND	Erosion of natural deposits
Total Uranium	No	6/04	30 ug/liter	0 ug/liter	ND - 0.48 ug/liter	Erosion of natural deposits

*** New York State Department of Health considers 50 pCi/liter to be the level of concern for beta particles.*

Microbiological Parameters	Violation Yes/No	Sample Date (or date of highest detected)	MCL	MCLG	Level Detected	Sources in Drinking Water
Total Coliform Bacteria	No[6]	8/08[7]	>5% of samples positive	NE	0.81% = highest percentage of monthly positives	Naturally present in environment
E. coli Bacteria	No[8]	NA	Any confirmed positive sample	0	No samples tested positive in 2008	Human and animal fecal waste

6 A violation occurs when more than 5% of the total coliform samples collected per month are positive.

7 In August 2008, two of the 247 samples taken in the distribution system indicated the presence of total coliform. Follow-up sampling, testing and reporting were performed as required, and the results were negative for both total coliform & E. coli.

8 A violation occurs when a total coliform positive sample is positive for E.coli and a repeat total coliform sample is positive or when a total coliform positive sample is negative for E.coli but a repeat total coliform sample is positive and the sample is also positive for E.coli.

During 2008, a total of only four samples tested positive for total coliform out of a total of 4,951 drinking water samples that were analyzed. Follow-up sampling, testing and reporting were performed as required by regulation, and the results were negative for both total coliform & E.coli in all cases. Since total coliforms were detected in less than 5% of the samples collected during any one month, the water system did not have any MCL violations. It should also be noted that E. coli, was not detected in any of these samples.

Giardia and Cryptosporidium	Violation Yes/No	Sample Date (or date of highest detected)	Number of Samples Testing Positive		Number of Samples Tested
			Giardia	Cryptosporidium	
Source Water	No	3/08	3	0	21
Treated Drinking Water	No	NA	0	0	21

Cryptosporidium is a microscopic pathogen found in surface waters throughout the United States, as a result of animal waste runoff. It can cause abdominal infection, diarrhea, nausea, and abdominal cramps if ingested. Our filtration process effectively removes Cryptosporidium. No Cryptosporidium was detected in any samples taken in 2008.

Giardia is a microbial pathogen present in varying concentrations in many surface waters. In 2008 Giardia was detected in 3 of 21 raw source water samples but was not detected in any treated drinking water samples. Giardia is removed/inactivated through a combination of filtration and disinfection or by disinfection alone.

UNREGULATED SUBSTANCES

Parameter	MCL	MCLG	Level Detected (mg/liter)	Range (mg/liter)
Alkalinity	NR	NE	91	55 - 133
Calcium Hardness	NR	NE	94	73 - 140
Total Dissolved Solids	NR	NE	158	144 - 173
Total Organic Carbon	NR	NE	2.1	1.63 - 3.85

ABBREVIATIONS AND TERMS

AL = Action Level: the concentration of a contaminant which, when exceeded, triggers treatment or other requirements which a water system must follow.

CFU/100 ml = Colony Forming Units per 100 milliliters

MCL= Maximum Contaminant Level: the highest level of a contaminant allowed in drinking water.

MCLG = Maximum Contaminant Level Goal: the level of a contaminant in drinking water below which there is no known or expected risk.

MFL = Million fibers/liter (Asbestos)

mg/liter = milligrams per liter (parts per million)

MRDL = Maximum Residual Disinfectant Level : the highest level of a disinfectant allowed in drinking water. There is convincing evidence that addition of a disinfectant is necessary for control of microbial contaminants.

MRDLG = Maximum Residual Disinfectant Level Goal: the level of a drinking water disinfectant below which there is no known or expected risk to health. MRDLGs do not reflect the benefits of the use of disinfectants to control microbial contamination

mrem/yr = millirems per year

ND = Not Detected: absent or present at less than testing method detection limit.

ng/liter = nanograms per liter = parts per trillion

NE = Not Established

NR = Not Regulated

NTU = Nephelometric Turbidity Units

pCi/liter = picocuries per liter

RAA = Running Annual Average

SU = Standard Units (pH measurement)

TT = Treatment Technique: a required process intended to reduce the level of a contaminant in drinking water.

ug/liter = micrograms per liter (parts per billion)

Variances and Exemptions= State or EPA permission not to meet an MCL or a treatment technique under certain conditions.

< : Denotes Less Than

≤ : Denotes Less Than or Equal To

The seal of the Partnership for Safewater as seen on this document indicates that we are part of a select group of water systems nationwide who have voluntarily committed themselves toward a proactive approach to strengthen the safety of drinking water for our customers above and beyond the current regulatory requirements. For additional information on the Partnership for Safewater visit www.awwa.org/science/partnership.

Results are from 2008 analyses or from the most recent year that tests were conducted in accordance with regulatory requirements. Some tests are not required to be performed on an annual basis. Information can be obtained upon request from the ECWA Water Quality Laboratory (716) 685-8570 or on the Internet at www.ecwa.org.

As you can see by the tables, our system had no violations. We have learned through our testing that some contaminants have been detected; however, these contaminants were detected below the level allowed by the State.

COMPOUNDS OR ELEMENTS TESTED FOR BUT NOT DETECTED

2-Chlorotoluene	1,1,2-Trichloroethane	Dalapon	N-nitroso-dimethylamine (NDMA)
4-Chlorotoluene	1,2,3-Trichloropropane	Di(2-ethylhexyl) adipate	N-nitroso-di-n-butylamine (NDBA)
2,4-D	1,1,2-Trichlorotrifluoro-ethane	Di(2-ethylhexyl) phthalate	N-nitroso-di-n-propylamine (NDPA)
4,4'-DDE	1,2,4-Trimethylbenzene	Dibromomethane	N-nitroso-methylethylamine (NMEA)
DCPA monoacid degradate	1,3,5-Trimethylbenzene	Dicamba	N-nitroso-pyrrolidine (NPYR)
1,2-Dibromo-3-Chloropropane	Acetochlor	Dichlorodifluoromethane	Oxamyl (Vydate)
DCPA monoacid degradate	Acetochlor ethane sulfonic acid	Dieldrin	PCB 1016
1,2-Dibromoethane	Acetochlor oxanilic acid	Dinoseb	PCB 1221
1,2-Dichlorobenzene	Alachlor	Diquat	PCB 1232
1,3-Dichlorobenzene	Alachlor ethane sulfonic acid	EPTC	PCB 1242
1,4-Dichlorobenzene	Alachlor oxanilic acid	Endothall	PCB 1248
1,1-Dichloroethane	Aldicarb	Endrin	PCB 1254
1,2-Dichloroethane	Aldicarb Sulfone	Ethylbenzene	PCB 1260
1,1-Dichloroethylene	Aldicarb Sulfoxide	Free Ammonia	Pentachlorophenol
cis-1,2-Dichloroethylene	Aldrin	Glyphosate	Perchlorate
trans-1,2-Dichloroethylene	Antimony	Heptachlor	Phosphate
1,2-Dichloropropane	Atrazine	Heptachlor Epoxide	Pichloram
1,3-Dichloropropane	Benzene	Hexachlorobenzene	Propacchlor
2,2-Dichloropropane	Benzo(a)pyrene	Hexachlorobutadiene	Propoxur
1,1-Dichloropropene	Beryllium	Hexachlorocyclopentadiene	n-Propylbenzene
cis-1,3-Dichloropropene	Bromobenzene	Hexahydro-1,3,5-trinitro-1,3,5-triazine	Selenium
trans-1,3-Dichloropropene	Bromochloromethane	Isopropylbenzene	Silver
1,3-dinitrobenzene	Bromomethane	p-Isopropyltoluene	Simazine
2,4-Dinitrotoluene	Butachlor	Lindane	Styrene
2,6-Dinitrotoluene	n-Butylbenzene	Manganese	Terbacil
3-Hydroxycarbofuran	sec-Butylbenzene	Mercury	Tetrachloroethylene
1-Napthol	t-Butylbenzene	Methiocarb	Thallium
2,3,7,8-TCDD (Dioxin)	Cadmium	Methomyl	Toluene
2,4,5-TP (Silvex)	Carbaryl	Methoxychlor	Toxaphene
1,1,2-Trichloroethane	Carbofuran	Methyl t-butyl ether (MTBE)	Trichloroethylene
2,4,6-trinitrotoluene	Carbon Tetrachloride	Methylene Chloride	Trichlorofluoromethane
2,2',4,4'-tetrabromodiphenyl ether	Chlordane	Metolachlor	Vinyl Chloride
2,2',4,4',5-pentabromodiphenyl ether	Chlorobenzene	Metolachlor ethane sulfonic acid(ESA)	Xylenes
2,2',4,4',5,5'-hexabromobiphenyl	Chloroethane	Metolachlor oxanilic acid (OA)	Zinc
2,2',4,4',5,5'-hexabromodiphenyl ether	Chloromethane	Metribuzin	
2,2',4,4',6-pentabromodiphenyl ether	Chromium	Molinate	
1,1,1,2-Tetrachloroethane	Copper	Napthalene	
1,1,2,2-Tetrachloroethane	Cyanide	Nitrite	
1,2,3-Trichlorobenzene	DCPA Diacid degradate	Nitrobenzene	
1,2,4-Trichlorobenzene	DCPA Monoacid degradate	N-nitroso-diethylamine (NDEA)	

New York State Department of Health Source Water Assessment

The New York State Department of Health completed a draft Source Water Assessment of the supply's raw water sources under the state's Source Water Assessment Program (SWAP). The purpose of this program is to compile, organize, and evaluate information regarding possible and actual threats to the quality of public water supply (PWS) sources. It is important to note that source water assessment reports estimate the potential for untreated drinking water sources to be impacted by contamination. These reports do not address the safety or quality of treated finished potable tap water. The Great Lakes' watershed is exceptionally large and too big for a detailed evaluation in the SWAP. General drinking water concerns for public water supplies, which use these sources include: storm generated turbidity, wastewater, toxic sediments, shipping related spills, and problems associated with exotic species (e.g. zebra mussels – intake clogging and taste and odor problems). The SWAP is based on the analysis of the contaminant inventory compiled for the drainage areas deemed most likely to impact drinking water quality at this public water supply's raw water intakes. Separate assessments were completed for the Lake Erie source and the Niagara River source. The assessment found a moderate susceptibility to contamination for the Lake Erie source. The amount of agricultural land in the assessment area results in elevated potential of disinfection byproduct precursors and pesticides contamination. While there are some facilities present, permitted discharges do not likely represent an important threat to source water quality based on their density in the assessment area. There is also noteworthy contamination susceptibility associated with other discrete contaminant sources, and these facility types include: landfills. The assessment found an elevated susceptibility to contamination for the Niagara River source. The amount of agricultural (and to a lesser extent residential) lands in the assessment area results in elevated potential for microbials, disinfection byproduct precursors, and pesticides contamination. There is also a high density of sanitary wastewater discharges, which results in elevated susceptibility for all contaminant categories. Non-sanitary wastewater discharges may also contribute to contamination. There is also considerable contamination susceptibility associated with other discrete contaminant sources, and these facility types include: chemical bulk storage, inactive hazardous waste sites, landfills, Resource Conservation and Recovery Act facilities and Toxics Release Inventory facilities.

If you have any questions about New York State's Source Water Assessment Program, please contact Ms. Dolores Funke, P.E., Senior Public Health Engineer, Erie County Health Department at 858-6966.
